# Transfer Learning and Machine Learning for Training Five-Year Survival Prognostic Models in Early Breast Cancer: Development and Validation Study

**DOI:** 10.2196/88665

**Published:** 2026-04-14

**Authors:** Lisa Pilgram, Kai Yang, Ana-Alicia Beltran-Bless, Gregory R Pond, Lisa Vandermeer, John Hilton, Marie-France Savard, Andreanne LeBlanc, Lois Shepherd, Bingshu Chen, John MS Bartlett, Karen J Taylor, Jane Bayani, Sarah Barker, Melanie Spears, Cornelis JH van der Velde, Elma Meershoek-Klein Kranenbarg, Luc Dirix, Elizabeth Mallon, Annette Hasenburg, Christos Markopoulos, Lamin Juwara, Fida K Dankar, Mark Clemons, Khaled El Emam

**Affiliations:** 1 School of Epidemiology and Public Health Faculty of Medicine University of Ottawa Ottawa, ON Canada; 2 Research Institute Children's Hospital of Eastern Ontario Ottawa, ON Canada; 3 Department of Nephrology and Medical Intensive Care Charité - Universitaetsmedizin Berlin Berlin Germany; 4 Division of Medical Oncology Faculty of Medicine University of Ottawa Ottawa, ON Canada; 5 Department of Oncology McMaster University Hamilton, ON Canada; 6 Cancer Therapeutics Program Ottawa Hospital Research Institute Ottawa, ON Canada; 7 Ottawa Hospital Cancer Center Ottawa Hospital Research Institute Ottawa, ON Canada; 8 Division of Medical Oncology and Hematology CHUM Université de Montréal Montreal, QC Canada; 9 Canadian Canadian Cancer Trials Group Queen's University Kingston, ON Canada; 10 Edinburgh Cancer Research Institute of Genetics and Cancer University of Edinburgh Edinburgh, Scotland United Kingdom; 11 Diagnostic Development Ontario Institute for Cancer Research Toronto, ON Canada; 12 Department of Laboratory Medicine and Pathobiology University of Toronto Ontario, ON Canada; 13 Department of Surgery Leiden University Medical Center Leiden, South Holland The Netherlands; 14 St. Augustinus Hospital Antwerp Belgium; 15 Department of Pathology NHS Greater Glasgow and Clyde Glasgow, Scotland United Kingdom; 16 Department of Gynecology and Obstetrics University Center Mainz Mainz Germany; 17 Medical School National and Kapodistrian University of Athens Athens, Attica Greece

**Keywords:** prognostic models, transfer learning, ensembles, breast cancer survival, machine learning

## Abstract

**Background:**

Prognostic information is essential for decision-making in breast cancer management. In recent years, trials and clinical practice have emphasized genomic prognostication tools, despite clinicopathological methods being more affordable and accessible. PREDICT v3 is one such tool with promising results across cohorts. Advances in machine learning (ML), transfer learning, and ensemble methods provide opportunities to enhance these approaches, especially where missing data and model assumptions differ across diverse populations.

**Objective:**

This study evaluates the potential to improve survival prognostication in breast cancer. More precisely, we compare de novo ML, transfer learning from the pretrained prognostication model PREDICT v3, and a stacked ensemble approach.

**Methods:**

Data from the MA.27 trial (NCT00066573) were used for model training, with external validation on data from the Tamoxifen Exemestane Adjuvant Multinational trial (NCT00279448 and NCT00032136) and a US Surveillance, Epidemiology, and End Results cohort. Transfer learning was applied by re-estimating the parameters of the pretrained prognostic tool PREDICT v3. De novo ML included random survival forests and extreme gradient boosting, and the ensemble was implemented using weighted linear stacking of model predictions. Internal and external validation was assessed in terms of the integrated calibration index and discrimination. Shapley Additive Explanations values were used to explain model predictions and decision-curve analysis to facilitate the interpretation of performance differences.

**Results:**

Transfer learning, de novo random survival forest, and the stacked ensemble improved calibration in MA.27 over the pretrained model (integrated calibration index reduced from 0.042 in PREDICT v3 to ≤0.007) while discrimination remained comparable (AUROC increased from 0.738 in PREDICT v3 to 0.744-0.799). In decision-curve analysis, these approaches demonstrated consistently positive net benefit across clinically relevant thresholds, while PREDICT v3 lost net benefit beyond 7.5% predicted risk. Invalid PREDICT v3 predictions were observed in 23.8% to 25.8% of MA.27 individuals due to missing information. In contrast, ML models and the stacked ensemble predicted survival despite missing data. Across all models, patient age, nodal status, pathological grading, and tumor size had the highest Shapley Additive Explanations values, indicating their importance for survival prognostication. External validation in the US Surveillance, Epidemiology, and End Results cohort confirmed the benefits of transfer learning, RSF, and ensemble in terms of calibration while maintaining discrimination at comparable levels. In contrast, generalizability was limited in the Tamoxifen Exemestane Adjuvant Multinational trial, a cohort with a substantially different distribution of clinicopathological characteristics.

**Conclusions:**

This study demonstrates that transfer learning, de novo RSF, and a stacked ensemble can improve prognostication compared with the pretrained PREDICT v3, particularly in the presence of missing or uncertain inputs. Transportability may be limited in cohorts with different clinicopathological profiles, requiring local validation before clinical deployment. Ultimately, better survival estimation can provide more meaningful guidance in breast cancer care.

**Trial Registration:**

ClinicalTrials.gov NCT00066573; https://clinicaltrials.gov/study/NCT00066573, NCT00279448; https://clinicaltrials.gov/study/NCT00279448, NCT00032136; https://clinicaltrials.gov/study/NCT00032136

## Introduction

Breast cancer is among the most common types of cancer worldwide. In 2022, there were 2.3 million women diagnosed with breast cancer globally [1], typically in a nonmetastatic stage at diagnosis [2]. Such early diagnosis allows for a broad range of treatment options, including surgery, radiation therapy, endocrine therapy, chemotherapy, and targeted systemic therapies. Estimating survival probabilities can support informed decision-making, especially in scenarios where multiple treatment options are available. This makes both prognostic information (a patient’s survival) and predictive information (a patient’s benefit from treatment) [3] highly valuable to guide patient management.

A large variety of clinicopathological and genomic risk assessment tools have been proposed to assist in clinical decision-making [3,4]. Other tools, for example, RSClin (provided by Exact Sciences), have been developed to improve prognosis by incorporating both clinicopathological and genomic information [5].

Trials such as Microarray In Node negative Disease may Avoid Chemotherapy [6] confirmed the importance but also the challenge of breast cancer risk assessment tools. It demonstrated that patients classified as low risk by genomic prognostication had favorable survival outcomes without chemotherapy. However, patients classified as high risk by genomic prognostication did not necessarily benefit from chemotherapy, particularly in discordant cases where clinicopathological risk was. Similarly, the recently published Adjuvant Systemic Treatment for (ER)-Positive HER2-negative Breast Carcinoma in Women Over 70 trial showed that women aged 70 years and older who had a high risk by genomic prognostication did not benefit from the addition of adjuvant chemotherapy to endocrine therapy in terms of survival [7]. Both trials underscore the limitations of current genomic prognostication tools in predicting treatment benefit and reinforce the critical distinction between prognostic and predictive information [3,8].

Despite these findings, genomic testing and prognostication have become routine even for clinicopathologically low-risk patients, diverging from clinical guidelines recommendations and thereby potentially resulting in overtreatment [3,5-7,9]. Also, genomic testing can come with delayed treatment decisions and considerable costs, limiting its accessibility in resource-constrained health care settings [10].

Importantly, recent initiatives have focused primarily on genomic prognostication tools. This is true for the clinical trials mentioned above but also for updates of clinicopathological tools that are used in practice with genomic signatures, even though the added value was found to be modest [11]. There has been relatively less interest in the refinement of clinicopathological prognostication [9], even though such solutions are inexpensive, easily accessible, and can support clinical decision-making. Furthermore, machine learning (ML) and deep learning approaches have been explored to enhance performance in survival prognostication with promising results but limited applicability in practice [12-14] (see section 1.2 in Multimedia Appendix 1).

A compelling opportunity also lies in leveraging validated pretrained models such as PREDICT v3 [15] and adapting them to new datasets [16]. In transfer learning, the idea is that a new task can be more effectively learned by transferring knowledge from a related task that has already been learned. There are multiple transfer learning approaches (see section 1.3 in Multimedia Appendix 1). Among them, parameter-based transfer learning (ie, fine-tuning) is the most useful as it does not require access to the original training datasets but instead fine-tunes the parameters of the pretrained model to the new data [16]. While such transfer learning is not yet widely adopted in survival analysis, there are promising results from lung cancer and pancreatic adenocarcinoma survival prognostication [17,18].

Complementary to de novo ML and transfer learning, ensemble integration offers a way to account for model-specific strengths and limitations by combining multiple models, such as fine-tuned and de novo trained ones [19]. This can ultimately provide a robust prognostication framework, particularly in cases where missingness and model assumptions vary across cohorts.

This study aims to better understand the benefits of transfer learning from pretrained models, de novo ML, and a stacked ensemble in survival prognostication for patients with breast cancer. More precisely, using the MA.27 study population [20], we investigated the following research questions:

- Parameter-based transfer learning (ie, fine-tuning): Can fine-tuning the pretrained prognostic tool PREDICT v3 to the MA.27 dataset improve survival prediction performance compared to the pre-trained model alone?
- De novo ML: How do state-of-the-art ML models trained directly on the MA.27 dataset compare against the (fine-tuned) pretrained model PREDICT v3?
- Ensemble integration: Does a stacked ensemble of fine-tuned pretrained models and de novo ML models add benefit compared to either approach alone?
- Generalizability: Do the potential benefits from fine-tuning (ie, question 1), de novo ML (ie, question 2), and the stacked ensemble (ie, question 3) still hold in external cohorts?

The primary clinical use case of the proposed model is to provide information that can support clinicians and patients in clinical management. Clinicians already use survival probabilities in discussions on the potential benefit of adjuvant systemic treatments following primary surgery [6,21-24], and patients also find such information important for their decision-making processes [24,25]. Beyond treatment decisions, individualized risk estimates can also inform follow-up schedules [26].

This study is methodological in nature and aims to establish and evaluate modeling strategies that can form the foundation for developing and improving prognostic tools for such clinical use. The intended user of such a model depends on its presentation: it can be an informative tool for a clinician, or, if carefully embedded within appropriate explanatory and interpretative guidance, as a resource for patients to prepare for discussions with their care team.

Ultimately, better survival estimation can provide meaningful guidance in breast cancer management, supporting a more targeted, cost-effective, and personalized approach to breast cancer care.

## Methods

### Reporting Guidelines

This study was conducted and reported in alignment with the Consolidated Reporting Guidelines for Prognostic and Diagnostic Machine Learning Models [27]. A completed Consolidated Reporting Guidelines for Prognostic and Diagnostic Machine Learning Models checklist is provided in Multimedia Appendix 2.

### Data Sources

This study is a secondary analysis of existing datasets from the MA.27 clinical trial [20], the Surveillance, Epidemiology, and End Results (SEER) program [28], and the Tamoxifen Exemestane Adjuvant Multinational (TEAM) trial [29], focusing on the prediction of 5-year breast-cancer survival. These datasets, their primary purpose, and their use across the different stages of this study are summarized in Table 1.

**Table 1:** Data sources.

Data	Description	Total sample size (N)	Used for		
			Model development, n^a^	Internal validation, n	External validation, n
MA.27^b^ [20]	Phase 3 randomized clinical trial comparing adjuvant hormone therapies in postmenopausal, hormone receptor–positive breast cancer (enrollment 2003-2008)	7563	6049	1514	—^c^
SEER^d^ [28]	Population-based cancer registry, hormone receptor–positive breast cancer (diagnosed in 2003), selected to align with MA.27 eligibility	27,064	—	—	27,064
TEAM^e^ [29]	Phase 3 randomized clinical trial of adjuvant hormone therapies in postmenopausal hormone receptor–positive breast cancer (enrollment 2001-2006), previously described subcohort from the entire trial	3825	—	—	3825

^a^“n” refers to the size of the cohort that was used in our analyses.

^b^MA.27 was randomly split into model development (80%) and validation (20%) partitions using stratification by outcome. Due to integer rounding within strata, exact partition sizes differed slightly from the exact proportions.

^c^Not available.

^d^SEER: US Surveillance Epidemiology and End Results.

^e^TEAM: Tamoxifen Exemestane Adjuvant Multinational.

Detailed information on the trials, including recruitment procedures, data collection, and delivery of the intervention (ie, adjuvant hormone therapy), is available in the respective primary publications and is not repeated here.

### MA.27 Study Cohort

MA.27 was a phase 3 clinical trial conducted by the Canadian Cancer Trials Group [20] of 7576 postmenopausal women with early-stage hormone receptor–positive breast cancer between 2003 and 2008, which compared 2 aromatase inhibitors, exemestane and anastrozole, as adjuvant endocrine therapy. The trial did not find a statistically significant difference in distant disease-free and disease-specific survival between the two arms. For the purpose of prognostic modeling, the cohort was therefore analyzed as a single population.

We limited the cohort to patients who were followed up beyond the day of enrollment (ie, time-to-event > 0) for our study to avoid biasing survival estimates with events unrelated to breast cancer. This subcohort consisted of 7563 patients. For this subcohort, variables were selected from MA.27 that overlapped with the information required for PREDICT v3 (Table 2; section 2.4 in Multimedia Appendix 1). This ensured alignment with variables considered clinically relevant and broadly available in the context of breast cancer prognostication. The outcome (ie, event) was defined as breast cancer–related death within a 5-year observation interval, and time-to-event or follow-up time was considered in the survival analyses.

**Table 2:** Variables selected for 5-year survival prediction.

Variables^a^	Explanation
Age	Age in years at randomization
Positive nodes	The number of positive lymph nodes
Tumor laterality	Side of tumor manifestation: right-handed, left-handed, or bilateral
Estrogen receptor status	Positive or negative estrogen receptor status
Progesterone receptor status	Positive or negative progesterone receptor status
Tumor size	The maximum size of the tumor in mm
Tumor grade	The pathological grading of the tumor from 1 (well-differentiated) to 3 (poorly differentiated)
Radiotherapy	Whether or not radiotherapy was received
Chemotherapy	Whether or not adjuvant chemotherapy was received
Trastuzumab therapy	Whether or not trastuzumab was received

^a^These variables represent the overlap between variables in MA.27 and those required for PREDICT v3. The variables required for PREDICT v3 but not directly available were year of diagnosis, smoking status, human epidermal growth factor receptor 2 status, Ki-67 status, mode of detection, micrometastases in case of one positive node, mean heart dose in case of radiotherapy, the type of chemotherapy in case of chemotherapy and bisphosphonate use. Some of them were mandatory for survival prognostication, and therefore, assumptions were made based on the standard of care at that time. For details, see section 2.4 in Multimedia Appendix 1.

Characteristics of MA.27 are described using median and IQR values for continuous variables and counts with percentages for categorical variables; descriptive visualizations (Kaplan-Meier curve, boxplots, and stacked bar plots) were generated to characterize the training cohort prior to modeling.

### Data Management

MA.27 was highly imbalanced in terms of its outcome, meaning that there were only 187 (187/7563, 2.5%) recorded disease-related deaths across a median follow-up of 4.1 (IQR 3.6-4.8) years. We used 2 different rebalancing strategies: (1) random oversampling examples technique on a dataset level and (2) weighting techniques on an algorithm level to help with model training [30] (section 2.1 in Multimedia Appendix 1). We tested the effect of both strategies in model training but could not detect a beneficial effect, and therefore they were not considered in the main analyses (section 3.5 in Multimedia Appendix 1).

MA.27 further presented with missingness in some variables. However, the mechanism of missingness was unclear. A missing progesterone receptor status could be, for example, not missing at random if it reflected ambiguity in the pathological assessment, suggesting a potentially biologically meaningful pattern of missingness. However, it could also be missing at random if values were omitted due to documentation errors or data entry inconsistencies.

We conducted missingness analyses and explored model-based imputation for these variables in MA.27 (section 2.2 in Multimedia Appendix 1). Imputation did not meaningfully change model performance (section 3.3 in Multimedia Appendix 1). However, because a nonrandom missingness mechanism could not be ruled out, and imputation may introduce bias under such conditions, imputed data were not used for the primary analyses. Instead, we leveraged the abilities of a tree-based ML model to internally handle missing data via surrogate splits or default directions, as they can handle mixed types of missingness patterns [31].

As mentioned in Table 2 and detailed in section 2.4 in Multimedia Appendix 1, the overlap between the variables in MA.27 and those required for PREDICT v3 was not complete, such that some variable values were constructed based on the trial’s metadata and relevant background knowledge. For example, HER2 status was inferred from trastuzumab use, and endocrine therapy from the inclusion criteria of MA.27. For other variables, no reliable approximation was possible, such that a fraction of patients for whom PREDICT v3 could not estimate survival remained. Sensitivity analyses assessing the impact of these assumptions on model performance are described in section 2.4 in Multimedia Appendix 1 and reported in section 3.4 in Multimedia Appendix 1.

### Survival Models

PREDICT was originally fitted on 5232 breast cancer cases from the UK East Anglia Cancer Registration and Information Centre diagnosed between 1999 and 2003 [15], updated recently to PREDICT v3 with 38,909 patients diagnosed between 2000 and 2017 [32], and validated on several cohorts around the world [33,34]. However, MA.27 differs in certain aspects from these training and validation cohorts: MA.27 was collected in 2003, involved approximately 5 years of follow-up, included only postmenopausal patients with hormone receptor–positive breast cancer, and lacked some of the information required in PREDICT v3. This makes de novo ML and transfer learning particularly useful. Section 1 in Multimedia Appendix 1 gives supplemental background on commonly applied survival models, including PREDICT, de novo ML, and transfer learning.

Given the structure of the MA.27 dataset with predominantly categorical variables, high censoring, and limited sample size, we opted to use tree-based methods for ML survival modeling. Such models are good for handling categorical data, can internally account for mixed missingness types, and offer robust performance without the data demands or complexity of deep learning approaches.

More precisely, this study included the following models:

1. PREDICT v3: The pretrained survival model, a competing risks Cox survival model with fractional polynomial baseline cumulative hazards.
2. f-PREDICT v3: The pretrained survival model fine-tuned to MA.27 (ie, transfer learning).
3. Random survival forests (RSFs) [35]: An ensemble method tailored for survival analysis.
4. Extreme gradient boosting (XGB) [36]: A gradient boosting framework with a survival-specific loss function.
5. Ensemble [19]: A stacked ensemble integrating f-PREDICT v3, RSF, and XGB whereby final predictions are obtained as a weighted sum of the individual model predictions.

Fine-tuning in the context of this study refers to parameter-based transfer learning whereby the initial model parameters of PREDICT v3 were used as initialization values for re-estimating the regression coefficients on MA.27. This differs from deep-learning fine-tuning, where the term typically refers to adapting a learned representation through changes in multilayer network weights.

Details on our implementation are provided in section 2.3 in Multimedia Appendix 1.

### Performance Measurement, Decision Curve, and Model Explainability

The primary goal of this study was to predict 5-year breast cancer survival, as this represents an early and clinically relevant milestone to guide decision-making [6,25]. Accordingly, performance metrics focused on this time point.

In internal and external validation, we measured performance for 5-year-survival prediction by area under the receiver operating characteristic (ROC) curve (AUROC) and integrated calibration index (ICI), and provided ROC curves and calibration plots for that time point. AUROC reflects a model’s ability to distinguish between individuals who survive and those who do not (ie, discrimination). ICI is the average absolute difference between predicted survival probabilities and observed survival outcome, estimated from a smoothed calibration curve over the entire range of predictions [37]. All performance measurements accounted for the time-to-event nature of the data in order to obtain robust measures in the context of heavy censoring. For discrimination, inverse probability of censoring weighting was leveraged to adjust for right-censoring, whereby the Kaplan-Meier estimator was used to model the censoring distribution [38]. It was implemented via the R package *timeROC* (Blanche et al [39]). For calibration, the observed outcome was modeled by a hazard regression–based method as proposed by Austin et al [37].

In breast cancer survival prognostication [32,40] or more broadly in evaluating the improvement of the predictive ability of markers [41], changes in AUROC of 0.05 or less have sometimes been highlighted. However, our interpretation follows a literature review on interpreting AUROC in health care [42] whereby label changes were typically triggered by a change in AUROC of at least 0.1.

The interpretation of ICI is more challenging as no uniform guidance exists. In experiments by Austin et al [37,43], correctly specified models yielded ICI values below 0.0125 while incorrectly specified ones had higher values. Similarly, a difference of 0.01 in predicted probabilities was considered as relevant by a nontrivial portion (17.7%) of patients with early breast cancer [25]. However, clinical decision making is typically triggered by probability differences of 0.03 to 0.05 [4,25,40,44], and some authors consider predicted probabilities within 10% of the observed outcomes as well-calibrated [32]. We align our interpretation with clinical decision-making standards and consider models with an ICI below 0.03 as well-calibrated.

The predicted probabilities were pooled across 10 independent runs to plot ROC curves in the internal validation, and a diagonal dashed line was added to indicate the discrimination of a random guess. For calibration plots, the observed probabilities were divided into four quartiles based on their predicted probabilities. Both predicted and observed probabilities were trimmed to exclude extreme values beyond the 10th to 90th percentile, and mean predicted and observed survival probabilities were calculated in each quartile. Horizontal and vertical error bars are illustrated to reflect the SDs of these quartile-wise means. A diagonal dashed line was added to indicate perfect calibration. In the internal validation, predictions were pooled across 10 independent runs to plot calibration.

To facilitate interpretation of differences in calibration and discrimination, we conducted decision-curve analysis (DCA) for 5-year survival [45,46]. DCA evaluates the net benefit of using a risk model to trigger clinical decision-making at a given threshold probability *P*_t_, compared to two default strategies: intervening in all patients or intervening in none. We deliberately use the term intervention to avoid narrow interpretation: this may represent a treatment decision, intensified follow-up, or additional diagnostics. Individuals with predicted 5-year mortality risk greater than or equal to *P*_t_ are considered candidates for a hypothetical intervention. This intervention is assumed to reduce the event probability but may be unnecessary in individuals who would not experience the event.

Because the outcome was time-to-event, net benefit at 5 years was estimated using the Kaplan-Meier method within the strata defined by the threshold choices, in line with the study by Vickers et al [45] and implemented via the R package *dcurves* (Sjoberg et al [47]). For each threshold *P*_t_, individuals were classified as high risk if their predicted risk exceeded *P*_t_. The cumulative event probability within the high-risk group was estimated using Kaplan-Meier, from which true and false positives were derived. Net benefit was calculated as:

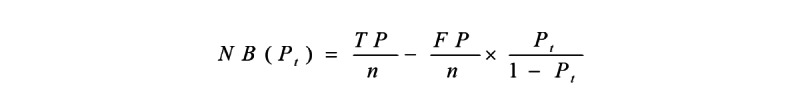



where *n* is the size of the cohort, and the weighting term 
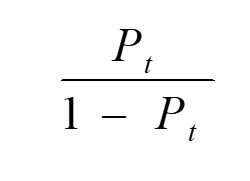
 reflects the relative costs of a false positive compared to the benefits of a true positive implied by the chosen threshold. Thresholds between 1% and 10% for 5-year mortality were evaluated, reflecting clinically plausible ranges in early breast cancer. The net benefit analysis was conducted in the internal validation cohort to illustrate how differences in calibration and discrimination can have decision implications.

Shapley Additive Explanations (SHAP) is a post hoc method to explain model predictions based on game theory [48]. For each individual prediction, a SHAP value can be assigned to each variable that reflects the contribution of that variable to the prediction. This value is estimated by a Monte Carlo approximation strategy as suggested in Štrumbelj et al [48]. A model-agnostic SHAP approach was leveraged to ensure consistent interpretation across the 5 different models. It was implemented via the R package *iml* (Molnar et al [49]). SHAP is presented for all individuals from MA.27 in a summary plot, implemented via the R package *shapviz* (Mayer et al [50]).

### Model Training, Testing, and Internal Validation

MA.27 was randomly split into three subsets: (1) 60% for training (data A), (2) 20% for testing (data B), and (3) 20% for final validation (data C).

**Figure 1:**
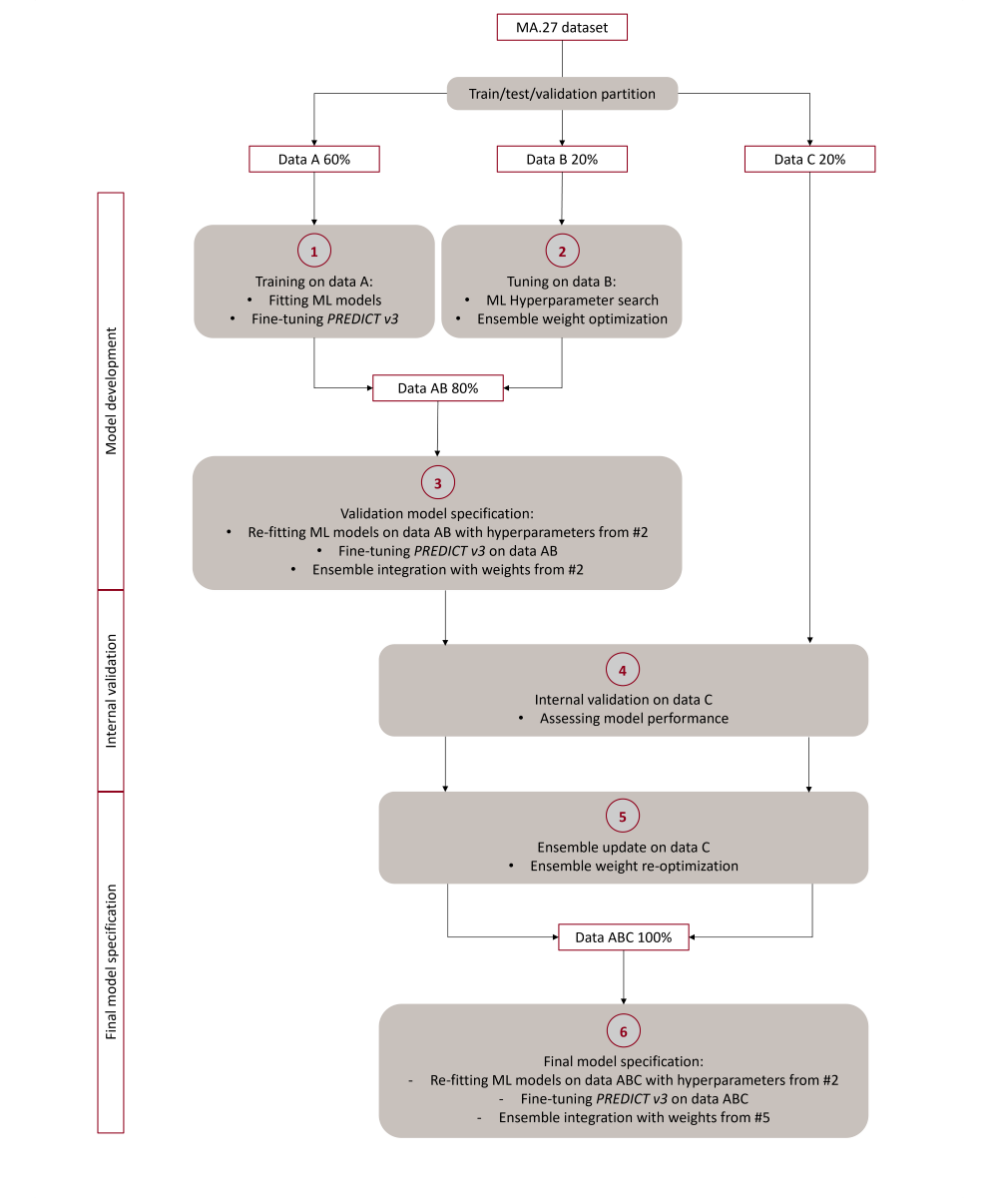
Model development, internal validation, and final model specification.

As shown in Figure 1, data A was used to train ML models and to fine-tune PREDICT v3 (ie, transfer learning). The fine-tuned model is referred to as f-PREDICT v3. Fine-tuning was performed by adjusting the 26 parameters of PREDICT v3 through a local optimization approach to MA.27 (section 2.4 in Multimedia Appendix 1).

Data B was used to find the best hyperparameters for the ML models and to determine the weights for stacking RSF, XGB, and f-PREDICT v3 as an ensemble (for details, refer to #2 in Figure 1). Details on hyperparameter and ensemble integration are provided in section 2.3 in Multimedia Appendix 1.

Data C was used as the hold-out set for internal validation. Prior to internal validation, the ML models were refitted on the combined data A and B using the best hyperparameters selected on data B, and PREDICT v3 was re–fine-tuned on the combined data A and B (for details, refer to #3 in Figure 1). The ensemble was constructed using the weights selected on data B, with the models refitted on the combined data A and B. These models were then evaluated alongside the original pretrained PREDICT v3 on the hold-out validation set (ie, data C).

After internal evaluation, the weights for the stacked ensemble were reoptimized using data C based on predictions from the models trained on the combined data A and B (for details, refer to #5 in Figure 1). Finally, the ML models were refitted on the full dataset (the combined data A, B, and C) using the previously selected hyperparameters, PREDICT v3 was re–fine-tuned, and the ensemble was constructed using the refitted models on the combined data A, B, and C and the optimized weights on data C (for details, refer to #6 in Figure 1).

During training, the ICI for 5-year survival prediction was used as the optimization goal. Calibration has clinically meaningful implications in prognostication tools as probability estimates typically guide decision-making. Discrimination (ie, AUROC), in contrast, focuses on ranking that may be more relevant for diagnostic tools. Other metrics, such as the mean absolute error, assess the accuracy of the predicted survival times, which, again, is different from our scenario where survival probabilities at certain time points are relevant for decision-making [51]. As a reference, an AUROC-based training approach was also conducted and is presented in section 3.6 in Multimedia Appendix 1. However, both calibration (ie, ICI) and discrimination (AUROC) were part of the evaluation.

To account for the variability in modeling across different partitions, all training, testing, and validation steps were repeated across 10 independent runs. The ML parameters were chosen by majority vote (ie, the parameter that was most often chosen across the 10 independent runs), the fine-tuned parameters for PREDICT v3, and the ensemble weights by averaging. We indicate the median and IQR values for the internal evaluation across the 10 independent runs.

To assess the robustness of the hyperparameter selection, we conducted a sensitivity analysis in which the variability of ML hyperparameters, of the fine-tuned PREDICT v3 parameters, and of the weights for the stacked ensemble was evaluated across a larger set of 50 independent runs. Results are reported in section 3.9 in Multimedia Appendix 1.

### External Validation

The final models were externally validated against a cohort from the US Surveillance, Epidemiology, and End Results program [28] and a clinical trial cohort (TEAM) with patients from Belgium, France, Germany, Greece, Japan, the Netherlands, the United Kingdom and Ireland, and the United States [52].

In SEER [28], the cohort was selected to match the relevant eligibility criteria of MA.27, namely hormone receptor–positive postmenopausal women diagnosed in 2003. It was also adjusted to meet the requirements of PREDICT v3, which should not be used in ductal carcinoma in situ or lobular carcinoma in situ only or in women with metastatic disease. Individuals with less than 1 day of follow-up were excluded, resulting in a final analytic cohort of 27,064 individuals.

We used data from a TEAM substudy [29]. TEAM, a trial of adjuvant endocrine therapy (exemestane vs tamoxifen followed by exemestane) in postmenopausal hormone receptor–positive breast cancer [52], had similar eligibility criteria as MA.27 . TEAM presented without a statistically significant difference in disease-free and overall survival between the two arms. For the purpose of prognostic survival modeling, the cohort was therefore analyzed as a single population. All individuals in TEAM had a nonzero follow-up time, such that the cohort was not further subselected. In total, 3825 individuals from TEAM were included in our analyses.

Information about missingness and variable mapping to PREDICT v3 for both of these external validation datasets is provided in section 2.4 in Multimedia Appendix 1. The external validation included calibration (ie, ICI) and discrimination (ie, AUROC) as detailed above. The 95% CI values were further derived from bootstrapping by taking the 2.5% and 97.5% percentile of the bootstrap distribution.

### Ethical Considerations

This project has been approved by the Ottawa Health Science Network Research Ethics Board (protocol ID 20210803-01H) and the Children’s Hospital of Eastern Ontario Research Ethics Board (protocol 25/107X). The Research Ethics Boards operate in compliance with, and is constituted in accordance with, the requirements of the Tri-Council Policy Statement: Ethical Conduct of Research Involving Humans; the International Conference on Harmonization Good Clinical Practice Consolidated Guideline; Part C, Division 5 of the Food and Drug Regulations; Part 4 of the Natural Health Products Regulations; and Part 3 of the Medical Devices Regulations and the provisions of the Ontario Personal Health Information Protection Act and its applicable regulations. This research involved the secondary use of health care datasets originally collected for purposes other than this study. This made the primary ethical consideration of this study the potential of disclosure risks. However, all datasets were deidentified at the source by the respective data custodians and were assessed as low risk. All analyses were conducted within a secure server environment with access restricted to authorized researchers of this study. These researchers have completed institutional privacy and security training, including instruction on the appropriate handling of personal health information, and, where required by data custodians, researchers also agreed to specific terms of use and completed additional ethics or data governance training. Individual re-consent was waived by the Research Ethics Boards, given that secondary use of deidentified data in this study posed minimal risk.

## Results

### Description of the MA.27 Study Participants

Our MA.27 dataset included 7563 postmenopausal women diagnosed with breast cancer. Table 3 provides an overview of the patients’ characteristics. Additional descriptive visualizations of the MA.27 cohort are provided in section 3.1 in Multimedia Appendix 1.

**Table 3:** Characteristics of the MA.27 study population.

Variables		Values^a^
Age, median (IQR)		64.2 (58.2-71.2)
**Nodal stage, n (%)**		
	N0	5360 (71.9)
	N1	1615 (21.7)
	N2	357 (4.8)
	N3	124 (1.6)
**Tumor laterality, n (%)**		
	Left-handed	3785 (50.1)
	Right-handed	3663 (48.4)
	Bilateral	115 (1.5)
**Hormone receptor status, n (%)**		
	Estrogen receptor status positive	7,513 (99.3)
	Progesterone receptor status positive	6079 (82)
Tumor size (cm), median (IQR)		1.5 (1-2)
**Tumor grade, n (%)**		
	Grade 1	1892 (32)
	Grade 2	2982 (50.5)
	Grade 3	1036 (17.5)
**Therapy, n (%)**		
	Radiotherapy	5370 (71.1)
	Chemotherapy	2326 (30.8)
	Trastuzumab therapy	67 (3.5)
Events, n (%)		187 (2.5)

^a^Percentages are calculated excluding missing values. The event was defined as breast cancer–related death within a 5-year observation interval. Details on missing values are provided in section 2.4 in Multimedia Appendix 1.

### Performance Across Transfer Learning, De Novo ML, and the Stacked Ensemble

We evaluated the prognostic performance of three potential improvement strategies, transfer learning or fine-tuning (f-PREDICT v3), de novo ML (RSF and XGB), and ensemble stacking, and compared them to the pretrained model PREDICT v3. This evaluation was done to identify the most effective strategy when conducting prognostication in a new cohort where the lack of certain information and distribution shifts can considerably compromise the performance of pretrained models.

In the following, we present the evaluation of the pretrained model itself as well as transfer learning, de novo ML, and the stacked ensemble when training was optimized for ICI without rebalancing and without imputation of missing values. Results for different rebalancing strategies and detailed missingness analyses are provided in section 3 in Multimedia Appendix 1.

Table 4 gives the summary of calibration (ie, ICI) and discriminative performance (AUROC) in the form of the median across the 10 independent runs as explained in the Methods section. In terms of calibration, fine-tuning (ie, f-PREDICT v3) and RSF presented the best results in the internal evaluation (ICI 0.005 and 0.003, respectively). Performance in terms of discrimination ranged from an AUROC of 0.738 (PREDICT v3) to an AUROC of 0.799 (f-PREDICT v3). Ensemble stacking of f-PREDICT, RSF, and XGB yielded results comparable to the best stand-alone models (ICI 0.007 and AUROC 0.746).

**Table 4:** Calibration and discrimination for 5-year survival.

Model^a^	Calibration (ICI^b^), median (IQR)	Discrimination (AUROC^c^), median (IQR)
PREDICT v3^d^	0.042 (0.039-0.047)	0.738 (0.719-0.770)
f-PREDICT v3^e^	0.005 (0.004-0.010)	0.799 (0.789-0.818)
RSF^f^	0.003 (0.002-0.008)	0.744 (0.731-0.760)
XGB^g^	0.040 (0.038-0.043)	0.783 (0.764-0.810)
Ensemble^h^	0.007 (0.003-0.009)	0.746 (0.733-0.766)

^a^Values were calculated on the validation dataset. Median and IQR values across 10 seed settings are indicated for all survival models. Training was done on the nonimputed and non-rebalanced dataset and optimized for the integrated calibration index.

^b^ICI: integrated calibration index.

^c^AUROC: area under the receiver operating characteristic.

^d^PREDICT v3: pretrained survival model.

^e^f-PREDICT v3: pretrained survival model fine-tuned to MA.27.

^f^RSF: random survival forest.

^g^XGB: extreme gradient boosting.

^h^Ensemble: stacked ensemble integrating f-PREDICT v3, random survival forest, and extreme gradient boosting.

**Figure 2:**
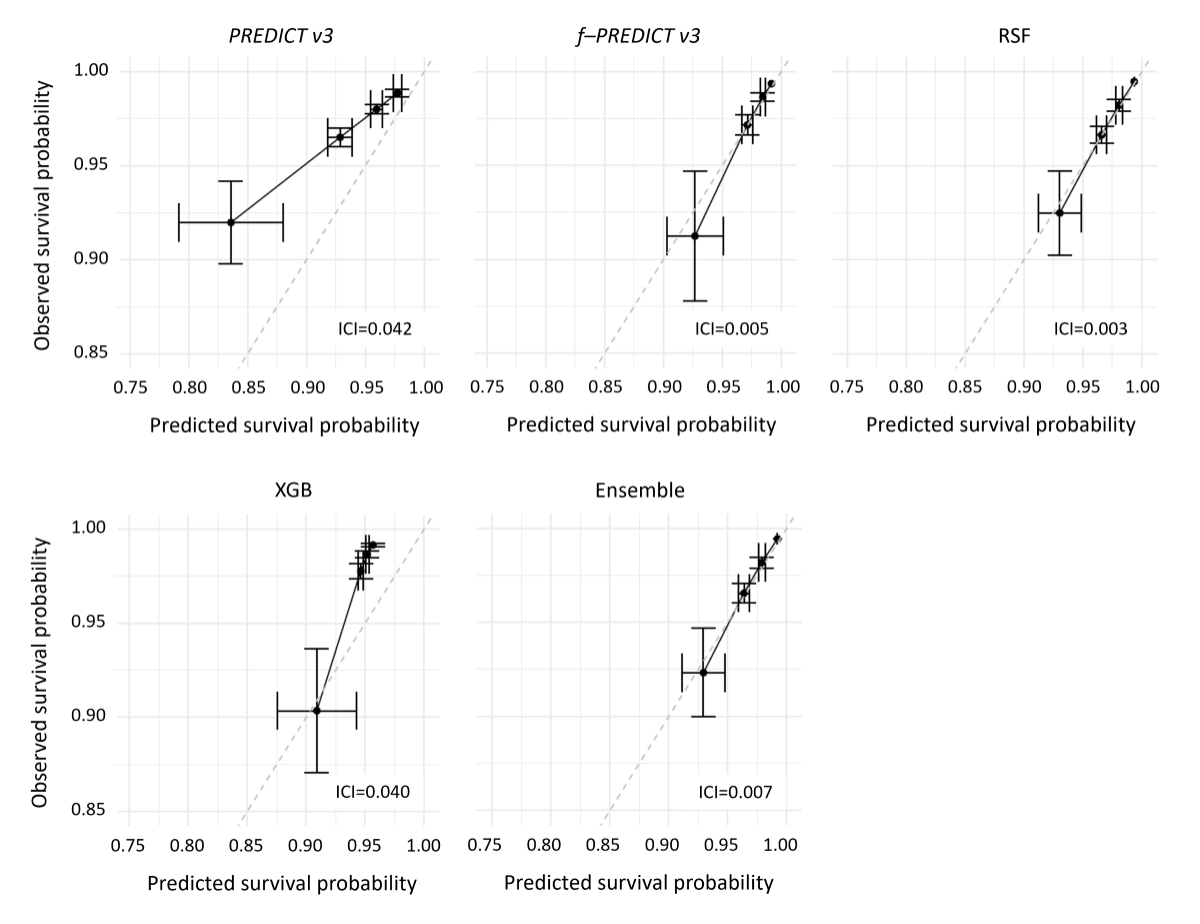
Calibration plots for 5-year survival. ICI: integrated calibration index.

**Figure 3:**
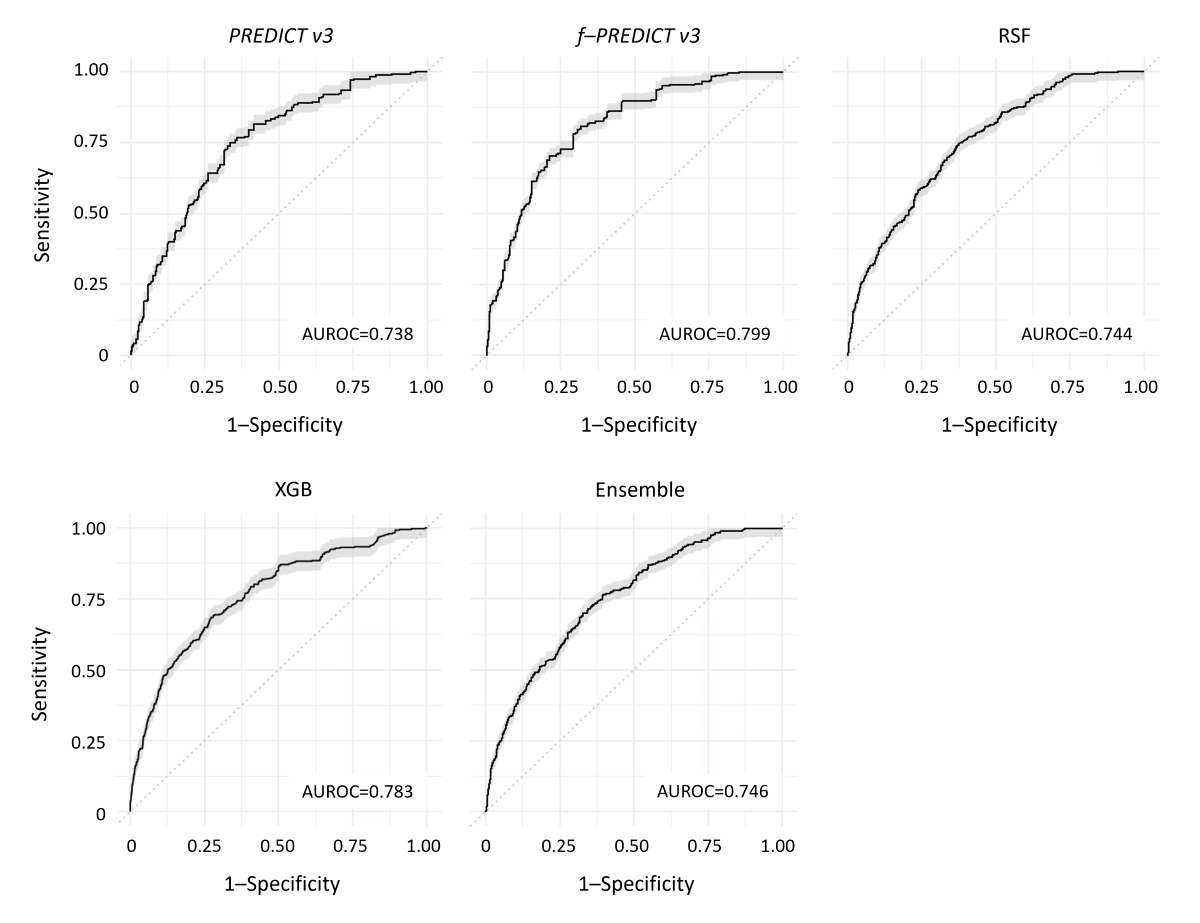
Receiver operating characteristic curves for 5-year survival. AUROC: area under the receiver operating characteristic curve.

Consequently, all three improvement strategies added value compared to the pretrained model PREDICT v3 in MA.27. Calibration plots are illustrated in Figure 2 for all survival models, with the diagonal dashed line indicating perfect calibration, and include the median ICI across the 10 independent runs for each model. ROC curves are shown in Figure 3, with the diagonal dashed line indicating discrimination of a random guess. The median AUROC across the 10 independent runs is given for each model. Both figures confirm the summary results.

MA.27 had incomplete records with respect to some variables that were required for PREDICT v3 for valid survival estimation. Consequently, PREDICT v3 and f-PREDICT v3 could not give survival estimates for a subset of the entire dataset when these mandatory variables were missing. We refer to these cases where PREDICT v3 and f-PREDICT v3 returned no estimate due to missing input variables as invalid predictions. Any results presented from PREDICT v3 or f-PREDICT v3 (eg, Figures 2 and 3) exclude these invalid predictions. The number of invalid predictions in PREDICT v3 and f-PREDICT v3 ranged from 361/1514 (23.8%) to 391/1514 (25.8%) across the 10 independent runs. In contrast, RSF, XGB, and the stacked ensemble could predict survival for all individuals, independent of missing information.

We provide performance evaluation stratified by whether or not PREDICT v3 and f-PREDICT v3 returned valid predictions in Table 5. In general, models performed worse in patients that were lacking relevant information, but these differences were very small, and RSF and the stacked ensemble still presented good calibration (ICI 0.014 and 0.015, respectively) in this subset.

**Table 5:** Calibration and discrimination in subsets with and without relevant missing information.

Model^a^		Calibration (ICI^b^), median (IQR)	Discrimination (AUROC^c^), median (IQR)
**Subset with valid predictions in PREDICT v3**			
	PREDICT v3^d^	0.042 (0.039-0.047)	0.738 (0.719-0.770)
	f-PREDICT v3^e^	0.005 (0.004-0.010)	0.799 (0.789-0.818)
	RSF^f^	0.006 (0.004-0.009)	0.763 (0.748-0.781)
	XGB^g^	0.043 (0.040-0.044)	0.802 (0.786-0.812)
	Ensemble^h^	0.006 (0.005-0.014)	0.775 (0.760-0.796)
**Subset with invalid predictions in PREDICT v3**			
	RSF	0.014 (0.013-0.015)	0.726 (0.713-0.746)
	XGB	0.051 (0.039-0.057)	0.745 (0.628-0.825)
	Ensemble	0.015 (0.015-0.021)	0.691 (0.668-0.739)

^a^Values were calculated on the validation dataset stratified by whether or not PREDICT v3 could provide survival estimates (ie, valid vs invalid predictions). Invalid predictions occurred in patients where relevant information was missing. Median and IQR values across 10 seed settings are indicated for all survival models. Training was done on the nonimputed and non-rebalanced dataset and optimized for the integrated calibration index.

^b^ICI: integrated calibration index.

^c^AUROC: area under the receiver operating characteristic.

^d^PREDICT v3: pretrained survival model.

^e^f-PREDICT v3: pretrained survival model fine-tuned to MA.27.

^f^RSF: random survival forest.

^g^XGB: extreme gradient boosting.

^h^Ensemble: stacked ensemble integrating f-PREDICT v3, random survival forest, and extreme gradient boosting.

### DCA

**Figure 4:**
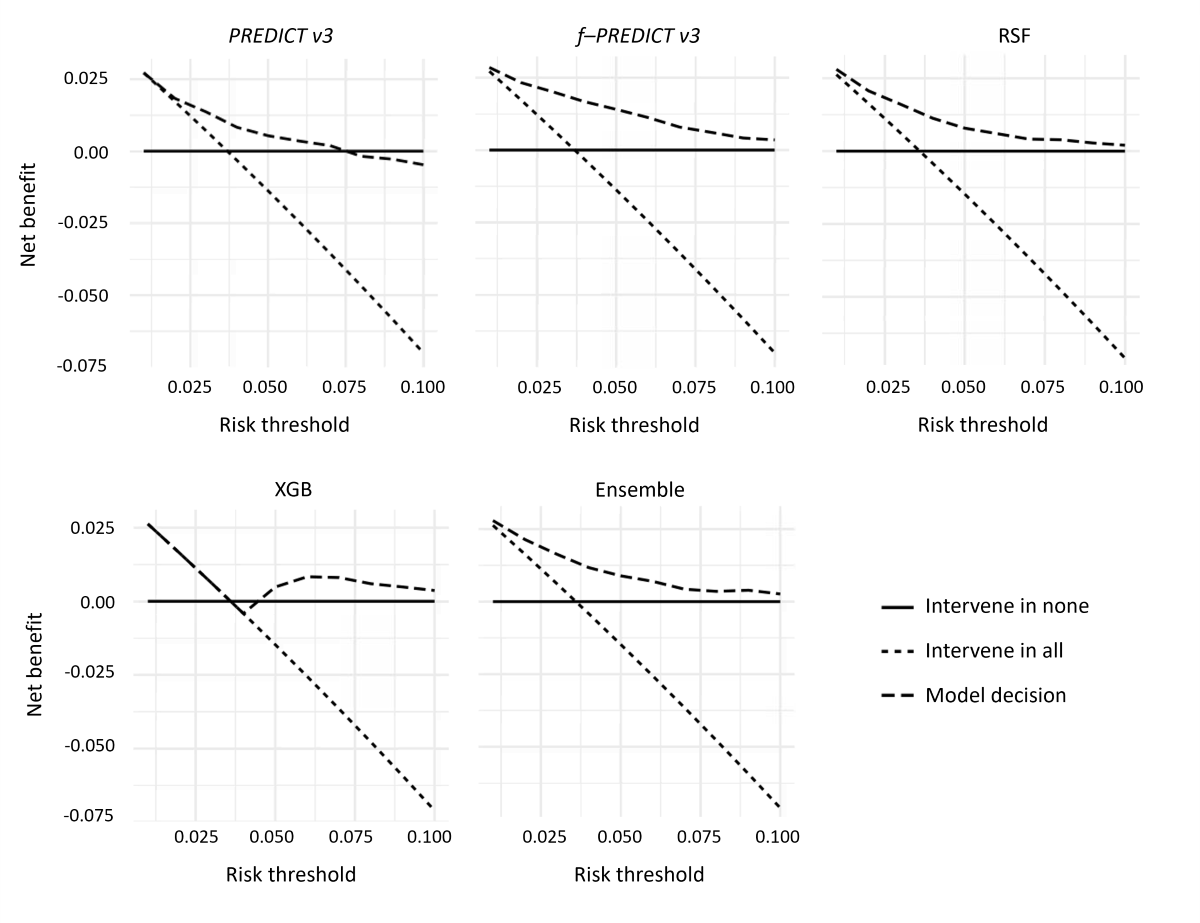
Decision-curve analysis for MA.27.

In Figure 4, results from the DCA are illustrated. More precisely, net benefit is plotted across risk thresholds (ie, predicted event probabilities) between 1% and 10%, and individuals with a predicted event probability (ie, risk) above the threshold were considered as intervention candidates. The DCA showed that f-PREDICT v3, RSF, and the ensemble yielded consistently positive net benefit across the evaluated threshold range, with the greatest benefit observed at lower thresholds. In contrast, PREDICT v3 crossed zero at an event probability of 7.5%, indicating that beyond this point, model-guided intervention was no longer preferable to intervening in none. Even at lower thresholds where PREDICT v3 was still net positive, its net benefit was consistently lower than that of f-PREDICT v3, RSF, and the stacked ensemble. XGB exhibited an unstable decision curve, consistent with its weaker calibration performance. At higher thresholds (ie, more than 8%-9%), net benefit approached zero for all models, reflecting the limited number of individuals exceeding such predicted event probabilities.

### Model Explainability

**Figure 5:**
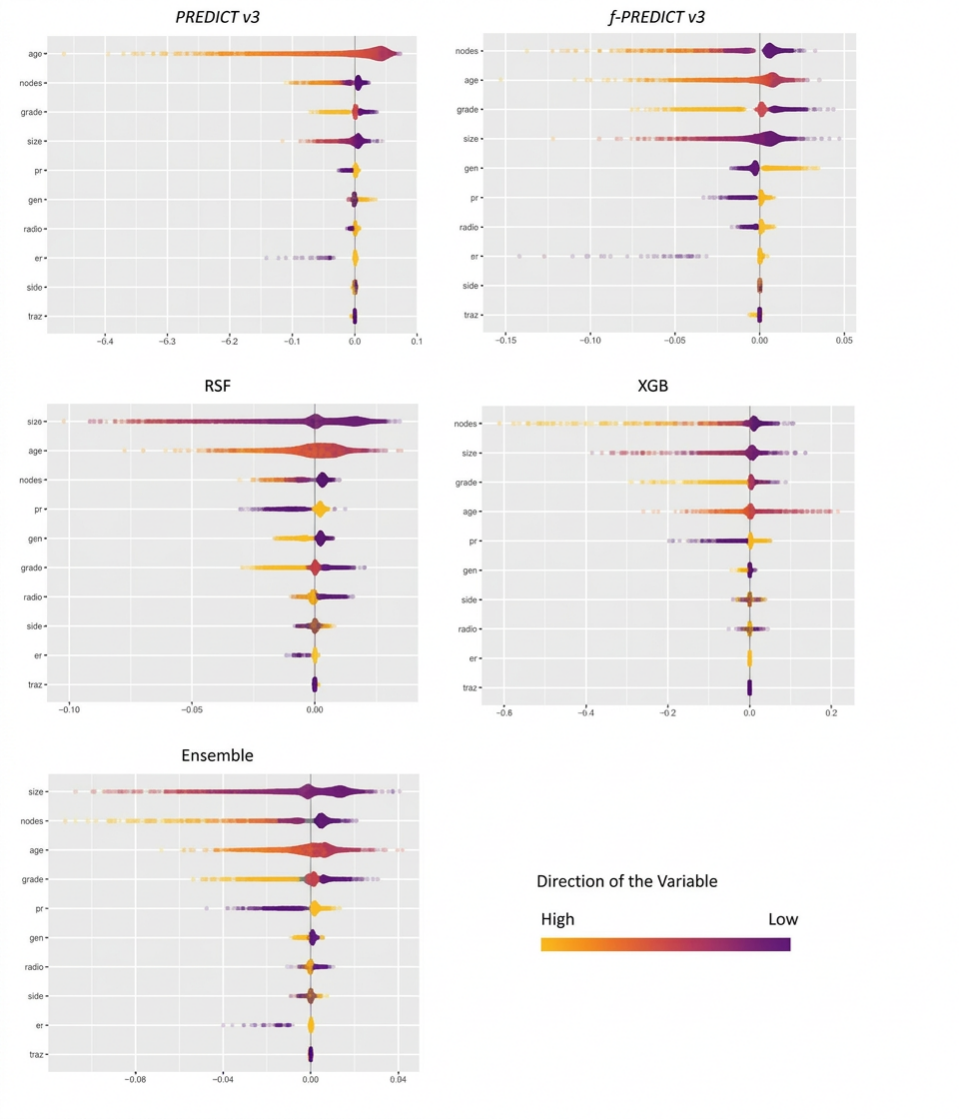
Shapley Additive Explanations analysis for MA.27. Er: estrogen receptor status; gen: chemotherapy; grade: pathological grading; nodes: number of positive nodes; pr: progesterone receptor status; radio: radiotherapy; side: tumor laterality; size: size of tumor; traz: trastuzumab therapy.

Results from the SHAP analysis are illustrated in Figure 5. SHAP values were thereby calculated for all records in MA.27, and the variables were ranked based on their mean absolute SHAP value across all observations, reflecting the importance of the variable for the entire MA.27 dataset. The *x*-axis indicates the SHAP value, the size reflects the strength of a variable’s contribution to the prediction, and the sign indicates the direction of this contribution (ie, positive SHAP values increase the survival probability). The direction of the variable is color-coded. While the exact top 3 variables in the SHAP analysis varied slightly between models, patient age, nodal status, pathological grading, and tumor size were consistently among them. In contrast, treatment information such as chemotherapy, radiotherapy, or trastuzumab was typically ranked less important across all models.

### External Validation on SEER and TEAM

To assess the extent to which the MA.27 model optimization was generalizable, we externally validated all models on data from the US SEER program and on the clinical trial dataset TEAM.

Results from the SEER cohort are shown in Table 6 and confirm the benefit of transfer learning, de novo ML, and the stacked ensemble over the pretrained model. Calibration was worse than in the internal evaluation with an ICI of 0.010 (f-PREDICT v3), 0.020 (RSF), and 0.018 (ensemble); discriminative performance was comparable to that in the internal evaluation. The bootstrap distribution of the ICI in PREDICT v3 and f-PREDICT v3 was strongly right-skewed, with most bootstrap estimates concentrated close to the point estimate (PREDICT v3 median 0.039, IQR 0.039-0.040, and f-PREDICT v3 median 0.012, IQR 0.011-0.013) but a small number of resamples producing extreme values, resulting in wider upper confidence bounds.

**Table 6:** Calibration and discrimination for US Surveillance, Epidemiology, and End Results data.

Model^a^	Calibration, ICI^b^ (95% CI)	Discrimination, AUROC^c^ (95% CI)
PREDICT v3^d^	0.039 (0.037-0.198)	0.765 (0.750-0.780)
f-PREDICT v3^e^	0.010 (0.009-0.173)	0.825 (0.811-0.838)
RSF^f^	0.020 (0.019-0.046)	0.753 (0.741-0.765)
XGB^g^	0.037 (0.034-0.093)	0.759 (0.747-0.771)
Ensemble^h^	0.018 (0.016-0.078)	0.792 (0.779-0.802)

^a^Values were calculated on the external validation data. The integrated calibration index and area under the receiver operating characteristic curve and their respective 95% CI values, as derived from bootstrapping, are indicated for all survival models. Training was done on the nonimputed and non-rebalanced MA.27 dataset and optimized for the integrated calibration index.

^b^ICI: integrated calibration index.

^c^AUROC: area under the receiver operating characteristic.

^d^PREDICT v3: pretrained survival model.

^e^f-PREDICT v3: pretrained survival model fine-tuned to MA.27.

^f^RSF: random survival forest.

^g^XGB: extreme gradient boosting.

^h^Ensemble: stacked ensemble integrating f-PREDICT v3, random survival forest, and extreme gradient boosting.

For TEAM, the respective results are given in Table 7. In contrast to SEER, the MA.27 optimized models performed worse on the TEAM dataset, with changes in AUROC up to 0.122 (XGB from 0.783 in internal evaluation to 0.661 in external validation via TEAM) and changes in ICI up to 0.074 with consistent overestimation of survival (f-PREDICT from 0.005 in internal evaluation to 0.079 in the external validation via TEAM).

**Table 7:** Calibration and discrimination for Tamoxifen, Exemestane Adjuvant Multinational data.

Model^a^	Calibration, ICI^b^ (95% CI)		Discrimination^c^, AUROC (95% CI)	
	Coefficient	95% CI	Coefficient	95% CI
PREDICT v3^d^	0.034	0.028-0.058	0.701	0.672-0.732
f-PREDICT v3^e^	0.079	0.072-0.089	0.707	0.677-0.736
RSF^f^	0.073	0.071-0.076	0.648	0.623-0.673
XGB^g^	0.061	0.051-0.091	0.661	0.635-0.687
Ensemble^h^	0.073	0.071-0.076	0.678	0.651-0.703

^a^Values were calculated on the external validation data. The integrated calibration index and area under the receiver operating characteristic curve and their respective 95% CI values, as derived from bootstrapping, are indicated for all survival models. Training was done on the nonimputed and non-rebalanced MA.27 dataset and optimized for the integrated calibration index.

^b^ICI: integrated calibration index.

^c^AUROC: area under the receiver operating characteristic.

^d^PREDICT v3: pretrained survival model.

^e^f-PREDICT v3: pretrained survival model fine-tuned to MA.27.

^f^RSF: random survival forest.

^g^XGB: extreme gradient boosting.

^h^Ensemble: stacked ensemble integrating f-PREDICT v3, random survival forest, and extreme gradient boosting.

To better understand the discrepancy between the external validation on SEER and TEAM, we conducted a stratified analysis within the SEER cohort (section 3.8 in Multimedia Appendix 1). When stratified by nodal status, tumor size, and tumor grade, model performance in SEER decreased within the subgroup defined by node-positive disease, tumor size ≥ 2 cm, and tumor grade G3. These characteristics were more prevalent in TEAM, and performance in this subgroup showed calibration and discrimination comparable to those observed in TEAM.

Calibration plots and ROC curves for both datasets are given in section 3.7 in Multimedia Appendix 1.

## Discussion

### Summary and Comparison With the Literature

This study investigated how innovative learning approaches, including pretrained models combined with transfer learning, de novo ML (RSF and XGB), and the stacked ensemble, can be leveraged to enhance the performance of prognostication tools for breast cancer. Using the MA.27 dataset, we addressed four questions: First, can fine-tuning the pretrained prognostic tool PREDICT v3 to the MA.27 dataset improve survival prediction performance compared to the pretrained model alone? Second, how do state-of-the-art ML models trained directly on the MA.27 dataset compare against the (fine-tuned) pretrained model PREDICT v3? Third, does a stacked ensemble using the fine-tuned pretrained model and de novo ML models add benefit compared to either approach alone? And fourth, do the potential benefits from fine-tuning, de novo ML, and the ensemble integration hold in external cohorts that are similar to MA.27?

All models, the pretrained PREDICT v3, the de novo ML (RSF and XGB), and the stacked ensemble, presented with moderate to good discrimination and calibration. The discriminatory ability of PREDICT v3 in our study was 0.738 (MA.27), 0.765 (SEER), and 0.701 (TEAM). This is worse than the published update by Grootes et al [32] using a cohort from the United Kingdom, where the AUROC for 5-year survival in hormone receptor–positive breast cancer ranged between 0.831 and 0.861. Chen et al [33] assessed PREDICT v3 in a Chinese cohort of 5424 women treated for nonmetastatic invasive breast cancer between 2010 and 2020, with an AUROC for 5-year survival in the hormone receptor–positive subcohort of 0.789. Hsiao et al [34] used SEER data from more than 860,000 patients (diagnosed between 2000 and 2018) to validate PREDICT v3 on US patients. In their study, the AUROC for 5-year survival was 0.797 in the hormone receptor–positive subcohort. Calibration was more difficult to compare directly between these studies due to differences in calibration metrics and presentation. However, visual comparison of calibration plots in the hormone receptor–positive subcohort suggests that PREDICT v3 was generally well-calibrated across these three studies [32-34]. If at all, there was a tendency to underestimate survival at the lower end of the survival prediction spectrum. In our study, this tendency was confirmed in MA.27 (ICI 0.042) and SEER (ICI 0.039) and appeared more pronounced than in the literature. TEAM, in contrast, showed an opposite pattern with a clear tendency to overestimate survival (ICI 0.034). While not directly comparable, a very recent validation of PREDICT v2.1 (not PREDICT v3) on Canadian patients with breast cancer diagnosed between 2004 and 2020 from Alberta achieved a more similar PREDICT v3 result to our study with an AUROC of 0.78 and an ICI of 0.03 (mixed cohort with hormone receptor–positive and hormone receptor–negative cancer) [53].

The lower performance of PREDICT v3 in our study and the one in Alberta reflects the challenge discussed in the introduction: MA.27 differs from the data used in the other studies, or more broadly, cohorts where pretrained models are deployed may diverge from the original training data.

Transfer learning (ie, f-PREDICT v3), de novo RSF (but not XGB), and the stacked ensemble outperformed the stand-alone pretrained PREDICT v3 in MA.27 and were similar in their performance. As model training was optimized for calibration, the improvement was most pronounced in the reduction of ICI (f-PREDICT v3 0.005, RSF 0.003, and ensemble 0.007 vs PREDICT v3 0.042 and XGB 0.040). The discriminatory ability also improved with an AUROC up to 0.799 (f-PREDICT v3), even though these differences would rather be considered negligible (less than 0.1 difference in AUROC). These findings were reflected in DCA, where improvements in calibration translated into consistent gains in net benefit across clinically relevant thresholds.

The external validation of the MA.27-trained models yielded mixed results: while model validation on the SEER cohort confirmed these findings, the benefit of transfer learning, de novo RSF, and the stacked ensemble did not apply to TEAM where the pretrained PREDICT v3 outperformed all alternative approaches with rather low calibration (ICI 0.039), and the MA.27-trained or fine-tuned models systematically overestimated survival on TEAM (ie, underestimated mortality), as reflected by calibration curves in section 3.7 in Multimedia Appendix 1. In other words, more 5-year breast cancer–related deaths occurred in TEAM than predicted by the models trained or fine-tuned on MA.27.

To better understand this discrepancy, we compared the cohorts in terms of their baseline characteristics. MA.27 and SEER were comparable across most patients’ characteristics, including age, nodal stage, tumor size, and tumor grade. In contrast, TEAM differed substantially in relevant prognostic variables: While MA.27 consisted predominantly of node-negative individuals (71.9%), only 39.3% of the TEAM cohort were node-negative [29]. Tumor size was also larger in TEAM compared to MA.27 (TEAM: 47.3% ≤2 cm, 46.8% 2-5 cm, 5.9% > 5 cm [29] vs MA.27: median 1.5, IQR 1-2 cm), and tumor grade was higher (TEAM: 11.7% grade 1 vs MA.27: 32% grade 1). Thus, TEAM represented a cohort with a more adverse clinicopathological risk profile (higher nodal burden, larger tumors, and higher grade).

These differences are likely directly relevant to the observed prediction error. A plausible explanation is a shift toward higher-risk disease in TEAM. Although nodal status, tumor grading, and tumor size were consistently among the most relevant variables for model predictions in MA.27 (see Model Explainability in Results section), the relationship between these variables and mortality was learned from a cohort in which high-risk profiles were relatively underrepresented. When applied to TEAM, which has a relative overrepresentation of such cases, the predicted survival decrement associated with these characteristics may have been insufficient. This interpretation is supported by a post hoc stratified analysis in SEER, in which model performance decreased to levels comparable to those observed in TEAM within a stratum characterized by node-positive disease, tumor size ≥ 2 cm, and tumor grade 3, with a similar pattern of survival overestimation (section 3.8 in Multimedia Appendix 1). These are characteristics that were more prevalent in TEAM than in SEER or MA.27.

To come back to the questions posed: first, fine-tuning the pretrained prognostic tool PREDICT v3 to the MA.27 dataset led to a substantial improvement in performance, particularly in terms of calibration but also in terms of discrimination, compared to the pretrained model alone. Second, state-of-the-art ML models trained directly on the MA.27 dataset have mixed results. RSF matches f-PREDICT v3 and outperforms the stand-alone pretrained model in terms of calibration but not in terms of discrimination; XGB showed the opposite pattern, with better discrimination but calibration closer to the stand-alone pretrained model. Third, the stacked ensemble did not add further benefit over either approach alone but, again, came with the advantage of providing predictions for all patients, even those with missing values, which is a practical advantage over f-PREDICT v3. And fourth, the observed benefits from fine-tuning and de-novo ML did extend to a similar SEER cohort. In this case, the ensemble appeared to be the best approach given its ability to handle missingness, its superior performance in both calibration and discrimination compared to PREDICT-v3, and its comparable performance to f-PREDICT v3. In contrast, none of these benefits generalized to the TEAM cohort, which is very likely due to the substantially different distribution of clinicopathological characteristics.

### Relevance of Missing Information

Missing information represents an important consideration in prognostic model development, evaluation, and real-world deployment. In this study, two distinct forms of missingness were relevant.

First, all cohorts exhibited record-level missingness, where variables were collected but incomplete for a subset of individuals. We report missingness patterns and analyzed the outcome association (section 3.5 in Multimedia Appendix 1). While we did not observe an association between missingness indicators and 5-year survival outcomes, we could not be certain about a random missingness mechanism given the original data collection context. Although sensitivity analyses using model-based imputed data showed performance results consistent with the nonimputed approach (section 3.5 in Multimedia Appendix 1), we refrained from using imputed data to avoid potential bias introduction under nonrandom missingness.

More importantly, record-level missingness limited the applicability of PREDICT v3 and f-PREDICT v3. Because these models require specific mandatory inputs, they could not generate survival estimates when certain variables were missing. This can affect a nontrivial portion of the overall dataset (23.8%-25.8% in MA.27). ML models, and the stacked ensemble come with the advantage of being able to generate predictions despite incomplete inputs. While the performance of RSF (ie, the best stand-alone ML model) was lower for these individuals, it still achieved discrimination comparable to the pretrained PREDICT v3 on individuals that PREDICT v3 could predict and demonstrated superior calibration.

Second, global missingness occurred when variables required by PREDICT v3 and f-PREDICT v3 were absent at the dataset-level rather than for specific records. In these cases, clinically informed assumptions were necessary to enable model predictions. Sensitivity analyses under optimistic and pessimistic assumptions (section 3.4 in Multimedia Appendix 1) showed that predictions from PREDICT v3 varied substantially under these assumptions, while f-PREDICT v3 was less sensitive, and the ML models and the stacked ensemble had no relevant variability across the different scenarios. This suggests that ML-based approaches and the stacked ensemble have greater robustness in settings where input variables must be approximated.

These findings are not only relevant for model development but also demonstrate that missing information can affect the operational functionality of prognostic tools in certain prediction settings.

### Addressing Outcome Imbalance

A particular methodological challenge of MA.27 was the low event rate. In binary classification tasks, such an imbalance is commonly addressed using dataset-level oversampling or algorithm-level weighted learning strategies [30]. The effectiveness of such strategies, however, varies across the literature, with differences across datasets and models [54-57].

In this study, we evaluated both approaches within a survival modeling framework. At the dataset level, a random oversampling examples approach [58] was applied. At the algorithm level, we implemented weighting during ML training. Neither approach improved overall performance. Dataset-level rebalancing via the random oversampling examples approach resulted in a substantial deterioration in calibration despite explicitly optimizing calibration during training. Algorithm-level weighting led to a comparatively less severe decline in performance. In contrast, models trained without outcome rebalancing achieved very good calibration (section 3.5 in Multimedia Appendix 1).

One possible explanation for this lack of benefit may lie in the differences between binary classification and survival modeling. In classification, imbalance directly affects the contribution of minority-class loss terms to the objective function. In contrast, survival models optimize more complex time-to-event objectives that incorporate both event occurrence and follow-up time. While recent methodological work has started to explore weight-based rebalancing within survival frameworks, particularly in Cox regression models [59], such approaches are not yet broadly adapted or standardized.

### Implications for Practice

In early breast cancer, survival prognostication is regularly used by clinicians and patients to inform discussions on adjuvant systemic treatment decisions, particularly chemotherapy, following primary surgery [6,21-25], and is also considered relevant for follow-up planning [26].

In this context, this study demonstrates that parameter-based transfer learning, de novo ML training, and ensemble stacking can help to improve prognostication of the widely used tool PREDICT v3 in situations where relevant information is lacking or a dataset shift is likely. Interestingly, these benefits can even generalize beyond the training cohort.

While missing information may not play a role in clinical prognostication where patients provide real-time information, it can still arise, for example, when clinicians are not sure about certain information and rely on clinical assumptions. Our analyses demonstrate that PREDICT v3 is very sensitive to such input assumptions, and considering alternative plausible input scenarios can help clinicians to better understand the potential variability of predictions.

Missing information is also commonly encountered when doing retrospective survival analyses. In such situations, de novo ML training and ensemble stacking can be good approaches for more reliable survival prediction. The stacked ensemble represents a particularly robust approach for prognostic modeling. It combines the strengths of transfer learning and de novo ML, maintains calibration and discrimination comparable to the best stand-alone models, generates predictions despite incomplete inputs, and remains stable under alternative assumption scenarios.

However, transportability of the stacked ensemble to cohorts with a higher patient risk profile may be limited (particularly node-positive, large, grade 3 tumors). To facilitate the identification of such cohorts, we deposited code in the Open Science Framework repository that reproduces the high-risk definition used in our analyses [60]. In such settings, local recalibration, re–fine-tuning, or re-training on cohorts more representative of the intended use population should be considered before clinical deployment. Future research may also explore stratified or “mixture-of-expert” ensembles, in which separate models are trained for clinically defined strata and integrated into an ensemble. This might potentially improve transportability across heterogeneous populations.

Beyond individual patient care, such models can be leveraged for emerging in silico trial designs by simulating counterfactual outcomes and enabling virtual comparisons of treatment effects [61-63].

### Limitations

This study has certain limitations, which are mainly driven by the dataset’s characteristics. There were some variables necessary for PREDICT v3 entirely missing in the dataset (eg, chemotherapy regimen in case of chemotherapy), such that assumptions were made where reasonably possible (see details in Multimedia Appendix 1). PREDICT v3 was sensitive to alternative assumptions and may perform better in situations where such assumptions are not necessary.

The follow-up time in MA.27 was limited to 5 years. While 5-year survival represents an early and clinically relevant milestone to guide decision-making, screening, and treatment developments have significantly improved outcomes in recent years, and therefore longer-term survival, such as 10- or 15-year survival, is becoming more relevant.

MA.27 is a randomized clinical trial. While trial data ensure standardized and high-quality data collection, participants in randomized trials may differ from the population, thereby limiting representativeness. However, external validation against registry data from SEER demonstrated good performance, supporting the applicability of our findings to broader, real-world patient populations.

Another limitation is that we did not account for the most recent update to PREDICT v4 [64]. This update refitted PREDICT v3 but did not incorporate further input variables. It has not yet been provided as a user-facing interface, making it less relevant for current clinical practice. More importantly, the validation presented for PREDICT v4 focuses on 10-year survival, whereas our analysis was restricted to 5-year survival. Direct comparison was therefore not possible, but the main results indicate that PREDICT v4 performs very similarly to PREDICT v3 with very small improvements in 10-year survival. We further note that the GitHub repository of the laboratory involved in the development of PREDICT provides another implementation (PREDICT v4.1.1) [65], for which no peer-reviewed model development or validation study has yet been published. This implementation includes ancestry as an additional covariate and allows optional incorporation of Oncotype DX recurrence scores. Because no peer-reviewed study has yet been published, the additional variables were not available in our cohorts, and, similar to PREDICT v4, no user-facing interface is currently available, this implementation was not included in our comparison.

The methodological work on model improvement in the presented study used the MA.27 clinical trial, which represents an older cohort. Nevertheless, the learnings would apply to more recent datasets, and the same methods can be applied to more recent cohorts to improve contemporary prognostic model performance.

It is also relevant to note that prognostic application is different from predictive one [3]. The good performance of our model in terms of survival prognostication does not imply that the model can estimate individual-level treatment benefits. Causal inference methods must be leveraged to better interpret such functionality.

## Data Availability

All original code for our analysis has been deposited in the Open Science Framework [60]. We used confidential health care data (MA.27 and TEAM) as well as accessible data from the US Surveillance Epidemiology and End Results for this study. Access to the US Surveillance Epidemiology and End Results can be requested through the National Cancer Institute’s SEER program.

## Appendix

Multimedia Appendix 1 Additional information.

Multimedia Appendix 2 CREMLS checklist.

## Abbreviations

AUROC: area under the receiver operating characteristic
DCA: decision-curve analysis
f-PREDICT v3: pretrained survival model fine-tuned to MA.27
ICI: integrated calibration index
ML: machine learning
ROC: receiver operating characteristic
RSF: random survival forest
SEER: US Surveillance Epidemiology and End Results
SHAP: Shapley Additive Explanations
TEAM: Tamoxifen Exemestane Adjuvant Multinational
XGB: extreme gradient boosting
