# Supplementary material for: Transfer Learning and Machine Learning for Training Five-Year Survival Prognostic Models in Early Breast Cancer: Development and Validation Study
Source: J Med Internet Res. 2026 Apr 14;28:e88665. doi: 10.2196/88665 (PMC13125978; doi:10.2196/88665)
Supplement: Multimedia Appendix 1 [file jmir_v28i1e88665_app1.pdf]

Supplementary Materials

Transfer Learning and Machine Learning for Training Five Year Survival Prognostic Models in Early Breast Cancer: Development and Validation Study

Appendix A

Contents

1. Supplemental Background 3
1.1 Traditional Survival Analysis Methods 3
1.2 Machine Learning in Survival Analysis 3
1.3 Transfer Learning in Survival Analysis 5
1.4 The Pre-Trained Model *PREDICT* in Breast Cancer Survival Analysis 5
2. Supplemental Methods 6
2.1 Outcome Re-Balancing 6
2.2 Missingness Analyses and Model-Based Imputation 7
2.3 Machine Learning Survival Models 7
2.3.1 Choice of Machine Learning Survival Models 7
2.3.2 Hyperparameter Tuning 8
2.4 Pre-Trained Model and Transfer Learning 8
2.4.1 The PREDICT v3 Model 8
2.4.2 Variable Mapping of MA.27, SEER and TEAM 9
2.5 Stacked Ensemble 17
3. Supplemental Results 17
3.1 Descriptive Characteristics of MA.27 17
3.2 Missing Data Patterns in MA.27 18
3.3 Model Performance Using Imputed Data 20
3.4 Model Performance Under Best and Worse Case Assumptions 21
3.5 Re-Balancing For Model Training 22
3.6 AUROC Optimization for Model Training 24
3.7 External Evaluation Plots 25
3.8 Stratified Results from External Validation on SEER 28
3.9 Parameter Variability Across 50 Independent Runs 30
4. References 33

1. Supplemental Background

1.1 Traditional Survival Analysis Methods

Modeling survival outcomes has a long history in statistics. The well-known Kaplan-Meier estimator [1] is one of the early foundational contributions to survival analysis and nonparametric statistics. It estimates the survival function directly from censored data, without any parametric assumption. The Kaplan-Meier estimator provides stepwise survival probabilities, allowing researchers to visualize survival functions and compare them across groups using statistical tests such as the log-rank test. However, it does not account for covariates, limiting its utility in multivariate analyses.

The Cox Proportional Hazards model [2], introduced under the proportional hazards assumption, represents a significant advancement as a semiparametric method to model survival outcomes. It estimates the hazard ratio associated with covariates while leaving the baseline hazard function unspecified. This flexibility allows the Cox model to handle complex survival data without distributional assumptions. However, the model relies on the proportional hazards assumption, which posits that the hazard ratios are constant over time. Violations of this assumption may limit its applicability in some datasets.

Parametric survival models have also been widely applied, leveraging specific assumptions about the distribution of survival times. Examples include the exponential, Weibull, log-normal, and log-logistic models [3]. These models provide precise estimates and enable extrapolation beyond the observed time range when the chosen distribution aligns with the underlying survival process. However, the parametric nature of these models makes them sensitive to misspecification of the distributional assumption.

Extensions of survival models include frailty models [4], which incorporate random effects to account for unobserved heterogeneity. Frailty models introduce a latent random variable, or frailty, that captures variability in survival outcomes not explained by observed covariates. Shared frailty models, for example, assume that individuals within the same group share a common frailty, leading to correlated survival times. These models are particularly useful for analyzing clustered data, where accounting for within-cluster dependence is crucial.

The Accelerated Failure Time (AFT) model [5] adopts a parametric approach to survival analysis by modeling survival time directly as a multiplicative function of covariates. In contrast to the Cox model, which focuses on hazard ratios, the AFT model examines how covariates accelerate or decelerate survival time. AFT models rely on assumptions about the distribution of survival times, commonly employing Weibull, log-normal, or log-logistic distributions. This approach provides an intuitive interpretation stemming from the distributional assumption, especially in many biological contexts where the timing of events is of primary interest. There is evidence that AFT models may naturally align with certain biological processes [6], particularly in scenarios where the proportional hazards assumption is violated.

1.2 Machine Learning in Survival Analysis

In recent years, the rapid advancement of machine learning (ML) research has led to the development of numerous approaches to predict survival outcomes. These methods leverage various algorithms to model survival data, accommodating censored observations and complex relationships among covariates. Among the prominent ML techniques adapted for survival analysis are tree-based methods, artificial neural networks, support vector machines, and Bayesian approaches.

Tree-Based Methods, such as Random Survival Forests [7] and gradient boosting trees like XGBoost [8], have been effectively extended to handle survival data. Random Survival Forests are a nonparametric tree-based ensemble learning method. At each node of a tree, the best split is determined based on survival-specific criteria, for example, the log-rank statistic. This criterion aims to maximize the difference

in survival probabilities between child nodes, thereby identifying the most informative splits for predicting survival outcomes. Similarly, gradient-boosting frameworks like XGBoost have been adapted for survival analysis by incorporating survival-specific loss functions. XGBoost extends gradient boosting trees using objectives based on the Cox proportional hazards model or accelerated failure time models.

Artificial Neural Networks, recognized as universal approximators [9,10], have been extensively applied in survival modeling to capture complex, nonlinear relationships and interactions among covariates. Various deep learning (DL) architectures have been adapted for survival analysis. For example, DeepSurv [11] extends the Cox proportional hazards model by employing a multilayer perceptron to model the hazard function. The network is trained by maximizing the Cox partial likelihood, allowing it to learn the underlying hazard relationships from data. DeepHit [12] combines DL with competing risks by modeling the joint distribution of survival times and event types. It utilizes a discrete-time hazard function and optimizes a loss function that accounts for both the event type and the time of occurrence. In addition, numerous approaches have been developed that use deep neural networks to model survival outcomes (see, for example, [13–18]). Despite their flexibility and strong predictive performance, neural networks in survival analysis present several challenges. They often lack interpretability, making it difficult to understand the influence of individual covariates on survival outcomes. Furthermore, neural networks are prone to overparameterization, which can lead to overfitting, especially in scenarios with limited sample sizes. Effective neural network training typically requires large datasets to capture the underlying patterns. In addition, the performance of neural networks can be highly sensitive to hyperparameter settings, initializations, and optimization algorithms, leading to variability in performance.

Support Vector Machines (SVM) have been adapted for survival analysis to handle time-to-event data by employing either ranking or regression constraints [19,20]. SVMs offer several advantages in survival analysis. They are particularly effective when applied to high-dimensional datasets and can capture complex, nonlinear relationships between covariates and survival outcomes through the use of kernel functions. However, SVMs have limitations. They lack straightforward interpretability of model parameters, similar to other kernel-based methods. The performance of SVMs is highly dependent on the choice of kernel and hyperparameters, necessitating careful tuning. Furthermore, SVMs can be sensitive to variations in input data, potentially leading to instability in predictions. Their effectiveness is also heavily impacted by censoring.

Bayesian Approaches to survival analysis offer a probabilistic framework that allows the incorporation of prior knowledge in the model parameters [21,22]. Bayesian methods offer several advantages, including the ability to incorporate prior knowledge, which is particularly useful in scenarios with limited data and prior knowledge. The Bayesian framework naturally provides credible intervals for model parameters and survival predictions, facilitating more comprehensive inferences. Additionally, Bayesian models are highly flexible, accommodating various types of censoring and extending to handle competing risks and time-dependent covariates. Bayesian methods come with several challenges. Careful selection of prior distributions is crucial to avoid bias, and inappropriate priors can lead to misleading inferences, especially in a data-scarce scenario. In addition, specifying appropriate models that accurately reflect the underlying survival mechanisms can be challenging and may require substantial domain expertise.

ML approaches have been increasingly developed for breast cancer prognostication in the past few years [23–25]. In 5-year survival prediction, these tools are most often based on SEER data with a dataset size of more than 10,000 patients [24]. Applied ML approaches are typically decision trees, neural networks and SVM and can present with promising discrimination. For example, in [26], RSF, SVM and XGB based survival models were compared against the Cox Proportional Hazards model. In this study, XGB outperformed the other models in terms of discrimination. Another study [27] reported comparable results in terms of discrimination between RSF and the Cox Proportional Hazards model. As shown by a

recent review, however, most studies with ML survival models lack a comprehensive performance evaluation including discrimination and calibration and are often not externally validated [24]. Also, various models treat survival as a binary classification problem thereby ignoring the time-to-event nature of that data [28,29]. These shortcomings may be an explanation for their limited applicability in clinical practice.

1.3 Transfer Learning in Survival Analysis

Transfer learning, similar to ML, has gained increasing attention in recent years. The idea is that a new task (i.e., target domain) can be more easily learned by transferring knowledge from a related task (i.e., source domain) that has already been learned [30]. In the context of DL and neural networks, transfer learning is particularly interesting as large datasets are required but not always available or accessible for training. This is very valuable in domains, such as healthcare, where resources are typically scarce and large datasets difficult to access [31–35]. There are multiple different approaches to transfer learning but most of them can be categorized into four groups: instance-based, feature-based, parameter-based or relational transfer learning [30]. Instance-based transfer learning re-weights the source data to align better with the target distribution, feature-based transfer learning re-weights the source variables or identifies a common latent representation for source and target variables, parameter-based transfer learning re-uses the parameters from a model trained on source data and relational transfer is the transfer via modeled relationships between source and target.

In survival analysis, as with other tasks in medical research, transfer learning can help with challenges such as heavy censoring or small sample sizes. Instance-based transfer learning for optimized survival analysis has been proposed, for example, in [36] where individuals were re-weighted to better align with the target distribution. Another instance-based example has been applied in [37] where data from biologically similar cancer entities was selected to improve survival models. However, access to the source data for instance re-weighting may not always be feasible due to privacy concerns or intellectual property limitations. Parameter-based transfer learning is a practical alternative in such scenarios. Instead of re-weighting the instances of the source data itself, the parameters of the pre-trained model are fine-tuned to the target data. In lung cancer survival prediction, for example, Zhu et al. fine-tuned a pre-trained DL prediction model on their target cohort and demonstrated its superiority against the model without fine-tuning [38]. In another example, survival analysis based on cancer transcriptomics data performed best in a DL approach with parameter-based transfer learning compared to more traditional models [39]. Similarly, parameter-based transfer learning added value in survival analysis of pancreatic ductal adenocarcinoma based on medical imaging [40].

1.4 The Pre-Trained Model *PREDICT* in Breast Cancer Survival Analysis

PREDICT is a tool for survival prognostication in breast cancer that has been widely adopted in practice. It was originally fitted on 5,232 breast cancer cases from the UK East Anglia Cancer Registration and Information Centre diagnosed in 1999-2003 [41], updated recently to PREDICT v3 with patients diagnosed between 2000 and 2017 [42] and validated on several cohorts around the world [43,44]. This version has been developed by Grootes, Wishart, and Pharoah [42] using a cohort from the UK. The total number of patients involved in development and validation exceeded 132,000. The model demonstrated robust discrimination (AUROCs between 0.78 and 0.82) and excellent calibration, with predicted mortality within 10% of the observed values, thus correcting the over-prediction observed with the earlier version (PREDICT v2.1).

Chen et al. [43] assessed PREDICT v3 in a Chinese cohort of 5,424 women treated for nonmetastatic invasive breast cancer between 2010 and 2020. Calibration was evaluated using quantile-based calibration graphs and chi-squared goodness-of-fit tests; The overall 5-year survival predicted by the

model differed from the overall survival observed by approximately −2% and nearly 0% in patients with ER negative. However, the model underestimated 5-year survival by approximately 9% in patients older than 75 years and by 5.8% in those with micrometastases, while it overestimated survival in patients who later developed distant metastases. Discrimination was similar to that of v2.2, with an overall AUROC around 0.756.

In [44], a US validation study using SEER data from more than 860,000 patients (diagnosed 2000–2018) examined PREDICT v3. Calibration was assessed by comparing observed versus predicted outcomes across risk quintiles, and the model's 10- and 15-year mortality estimates were within 5–8% of the observed values for both ER-positive and ER-negative patients. The AUROC values were approximately 0.769 for ER-positive and 0.738 for ER-negative cases at 10 years. Notably, subgroup analysis revealed that the model over-predicted mortality in non-Hispanic Asian patients with ER-negative disease and under-predicted mortality in non-Hispanic Black patients with ER-positive disease, suggesting that recalibration may be needed for these groups.

The earlier version, PREDICT v2.1, was also recently validated in a cohort from Alberta, Canada, diagnosed between 2004 and 2020 [45]. Despite being the earlier version, it achieved good discrimination with AUROC values of 0.78 for 5-year and 0.73 for 10-year survival. In comparison, an ML approach using Random Survival Forests demonstrated lower discrimination (0.67 and 0.64 respectively) and calibration.

More recently, results from a re-calibration of *PREDICT v3* to *PREDICT v4* have been published with reported small improvements in 10-year survival [46], but not yet provided as a user-facing interface, making it less relevant for current clinical practice. The GitHub repository of the laboratory involved in the development of *PREDICT* provides a further updated version (*PREDICT v4.1.1*) [47], for which no peer-reviewed model development or validation study has yet been published.

2. Supplemental Methods

2.1 Outcome Re-Balancing

Re-balancing strategies for imbalanced data can be broadly categorized into dataset-level techniques, where the training dataset is modified, or algorithm-level techniques, where the learning procedure itself is adjusted [48]. In this study, we evaluated both approaches in their ability to support model development.

At the dataset-level, the Random Over-Sampling Examples (ROSE) technique was applied which can ensure a more balanced and representative distribution of outcome [49]. For ROSE, missingness was encoded as dummy variable (in numerical variables) or as separate category (in categorical variables) to preserve its structure. This was reversed in a post-processing step. We implemented ROSE via the R library ROSE [50]. Results are presented in the supplemental results section below. The ROSE technique was used to re-balance the training dataset to support model development. It therefore affected all models trained or fine-tuned on MA.27, including the two de-novo ML models (see ML model details in **Appendix A: Supplemental Methods – Machine Learning Survival Models**) and the *f-PREDICT v3*.

At the algorithm-level, we implemented weighted re-balancing strategies during ML training, and the weight was treated as a hyperparameter. The weight was thereby defined in terms of the target event fraction. For Random Survival Forests, weights were incorporated via case-weighted bootstrap sampling [51], increasing the probability that event observations were selected into individual trees. For Extreme Gradient Boosting, weights were supplied through the training data matrix, resulting in the optimization of a weighted Cox partial likelihood.

Both approaches differ from class-weighted classifiers where misclassification errors are penalized under a binary loss function [48]. In contrast, survival models optimize time-to-event-specific objectives that account for both event occurrence and follow-up time. Consequently, while weighted re-balancing strategies can be implemented on an algorithm-level in survival modeling, their interpretation is different from classical cost-sensitive classification.

Results are presented in **Appendix A: Re-Balancing for Model Training**.

2.2 Missingness Analyses and Model-Based Imputation

Missingness data patterns were characterized for MA.27 and the two external cohorts, SEER and TEAM (see **Appendix A: Supplemental Methods – Variable Mapping of MA.27, SEER and TEAM**).

More in-depth analyses including the associations between missingness and survival outcomes as well as sensitivity analyses using imputed data were conducted on the training dataset MA.27. To assess whether missingness was informative with respect to survival outcomes, missingness indicators for each variable with non-zero missingness and univariable Cox proportional hazard models were constructed. Results are reported in **Appendix A: Supplemental Results – Missing Data Patterns in MA.27**.

Model-based imputation was applied via the R library mice [52]. We used random forests as imputation model and generated one imputed dataset. This procedure was explored for missing variables in MA27, but we ultimately refrained from its application since we could not rule out a non-random missingness pattern. Results are presented in **Appendix A: Supplemental Results – Model Performance Using Imputed Data**.

2.3 Machine Learning Survival Models

2.3.1 Choice of Machine Learning Survival Models

In light of the advantages and disadvantages described in the supplemental background, we opted to use tree-based methods to model survival in the MA.27 dataset. This decision is informed by several key characteristics of the data and the inherent strengths of tree-based algorithms.

1. Predominance of Categorical Covariates: The MA.27 dataset primarily consists of categorical covariates. Tree-based methods, such as Random Survival Forests and Gradient Boosting Machines, are inherently adept at handling categorical variables without extensive preprocessing.
2. High Proportion of Censored Observations and Limited Number of Observations: With over 95% of the patients not experiencing the event within the 5-year observation period and approximately 7000 data points in total, the dataset presents significant challenges and makes deep learning approaches less viable due to their data-intensive nature and complex parameterization needs.
3. Modeling Nonlinearity: Tree-based models effectively capture nonlinear relationships, provided they are not excessively complex. In the MA.27 dataset, the limited number of continuous variables minimizes concerns about nonlinearity. Therefore, methods such as support vector machines and neural networks, which are designed to handle more intricate nonlinear interactions, offer limited advantages in this context.
4. Natural Handling of Missing Data: Tree-based algorithms manage different missingness types as mentioned in the section on Data Management. This makes them also ideal for developing web-based prediction tools since they can seamlessly manage incomplete user provided data while ensuring robust predictions.

In summary, the decision to leverage tree-based methods for survival prognostication in the MA.27 dataset was underpinned by the dataset's characteristics. Random Survival Forests (RSF), an ensemble method tailored for survival analysis, and Extreme Gradient Boosting (XGB), an efficient and scalable implementation of gradient boosting machines with a survival-specific loss function, were leveraged in this study. RSF was implemented via the R package randomForestSRC [51]; XGB via the R package xgboost [53].

2.3.2 Hyperparameter Tuning

Hyperparameter tuning for these models was executed using a zero-order search methodology (grid search) to optimize model performance across all components using the MA.27 testing subset (i.e., data B). The following hyperparameters were considered:

	Hyperparameter	Explanation	Values considered
RSF	ntree	Number of trees in the RSF	500; 1000; 1500
	mtry	Number of variables sampled at each split	3; 4; 6
	nodesize	Minimum terminal node size	3; 5; 10; 15
	splitrule	Splitting criterion	logrank; logrankscore
	targetfrac	Target weighted event fraction in case of algorithm-level re-balancing	0.1; 0.2; 0.33
XGB	eta	Learning rate	0.05; 0.1
	max_depth	Maximum depths of trees	2; 5
	subsample	Subsample ratio of training instances	0.6; 1
	colsample_bytree	Subsample ratio of columns when constructing each tree	0.6; 1
	lambda_vals	L2 regularization coefficient on leaf weights	0.05; 0.1
	nrounds	Number of boosting rounds	500
	targetfrac	Target weighted event fraction in case of algorithm-level re-balancing	0.1; 0.2; 0.33

Table 1. Hyperparameters Considered in Grid Search When Tuning ML Survival Models.

2.4 Pre-Trained Model and Transfer Learning

2.4.1 The PREDICT v3 Model

PREDICT v3 is publicly available under [54]. We adjusted the code to flag potential contradictory inputs (e.g. HER2 therapy but HER2 negativity), automatically impute detection mode by age and screening

practices and infer mean heart dose by tumor laterality. The code was further vectorized to allow for more efficient calculation (the adjusted code is available in [55]).

To fine-tune *PREDICT v3* for the MA.27 cohort, we optimized its 26 parameters using the MA.27 training subset (i.e., parameter-based transfer learning). *PREDICT v3* takes the form of a competing risk Cox survival model with fractional polynomial baseline cumulative hazards [42]. Fine-tuning in this context corresponds to re-estimating the model's regression coefficients on MA.27 by maximizing the Cox partial likelihood. The initial parameters were thereby used as initialization values. The optimized version is referred to as *f-PREDICT v3.* The parameter search was conducted via the gradient-free optimization algorithm Nelder-Mead [56] which is more robust in settings where differentiability cannot easily be assumed. This is a local optimization approach and was implemented via the R package STAT [57].

2.4.2 Variable Mapping of MA.27, SEER and TEAM

PREDICT v3 requires certain information about the patient to predict their survival. While some missing variables can be handled internally by the model, the absence of certain key predictors prevents the tool from giving a valid survival estimate. It should further not be used in women with DCIS/LCIS only or in women with metastatic disease. The MA.27 and TEAM eligibility criteria were in line with these requirements; SEER data was selected to match these eligibility criteria.

MA.27 collected various information including demographic, clinicopathological and treatment-related variables but did not provide all of the necessary information at the desired level of granularity, or in some cases, at all. In **Table 2**, we give details about the *PREDICT v3* input variables and their availability in MA.27 and, if applicable, assumptions we could take based on background knowledge about the study population. **Table 3** and **Table 4** give these details for SEER and TEAM respectively.

While these assumptions represent the most clinically plausible choices given the study context, we additionally conducted sensitivity analyses under an optimistic and a pessimistic clinical scenario to assess the robustness of model performance to assumption uncertainty. These two scenarios were defined to be consistent with the directionality of effects encoded in the *PREDICT v3* model [58].

In the pessimistic scenario, the year of diagnosis was kept at 2003, all participants were assumed to be smokers, cancers were assumed to be detected symptomatically, and all tumors were assumed to be HER2 positive. For patients receiving chemotherapy, a standard anthracycline-based regimen was assumed, no trastuzumab was assumed despite HER2 positivity and no bisphosphonate use was indicated for all participants.

In the optimistic scenario, the year of diagnosis was set to the last year of recruitment (i.e., 2008), participants were assumed to be non-smokers, cancers were assumed to be detected by screening and tumors were assumed to be HER2 negative in cases without explicit or with negative trastuzumab treatment. For patients receiving chemotherapy, a taxane/high-dose anthracycline-based regimen was assumed. Trastuzumab was assumed not to be given in cases with missing information, consistent with the assumption of HER2 negativity in these individuals. Bisphosphonate use was assumed for all participants.

The results of these two scenarios are presented in **Appendix A: Supplemental Results – Model Performance Under Best and Worse Case Assumptions**.

Variable in *PREDICT v3*	Mapping to MA.27	Missingness
Year of Diagnosis	Year of diagnosis was unknown for participants in the MA.27 study. We assumed diagnosis to be close to enrollment since the treatment timeline for breast cancer patients is well established in clinical practice and the initiation of hormone therapy typically occurs within a few months following diagnosis and surgery. We therefore used the start of recruitment (i.e., 2003) as year of diagnosis. We did not have the precise year of enrollment and year has a positive effect on survival, so that we may have underestimated survival (see limitations in main manuscript).	100.0%
Age in Years	Age in years was available for all patients in MA.27.	0.0%
Postmenopausal Status	All patients in MA.27 were postmenopausal (see inclusion criteria [59]).	0.0%
Smoking Status	As smoking status is required by *PREDICT v3* but was not available in MA.27, we assumed smoker status as 0 (i.e., non-smoker). However, at the time of MA.27 (i.e., 2003), 21% of Canadians were current smokers [60] and smoking can influence treatment outcome (see limitations in main manuscript). We also trained a model based on the assumption of a positive smoker status for all patients and that did not exhibit any differences in performance measurements and therefore we decided to assume non-smokers.	100.0%
Estrogen Receptor Status	Information on estrogen receptor status was available for all patients in MA.27.	0.0%
Progesterone Receptor Status	Information on progesterone receptor status was not available for all patients in MA.27 and could not be inferred for those patients using background information. This information is not necessarily required by *PREDICT v3* but can be left as missing.	2.0%
HER2 Status	The MA.27 dataset did not provide details on the HER2 status but includes a variable, indicating Herceptin (trastuzumab) usage. This variable was introduced in 2005, following the publication of positive results showing efficacy of trastuzumab in HER2-positive early breast cancer patients. While we can assume that patients with trastuzumab usage are HER2 positive, the situation is more complex for patients who did not receive trastuzumab: from 2005 onwards, it is very likely that these patients are HER2 negative, as trastuzumab would have been part of their standard treatment, if they were positive. Before 2005, however, this assumption does not hold as trastuzumab was not widely used for HER2 positive patients. Since we did not have the year of diagnosis or enrollment, we could not make a distinction. While *PREDICT v3* accepts missing information as an input, we decided to assume HER2 negativity for patients where information about trastuzumab was not indicated (see limitations in main manuscript). This may better reflect clinical reality since around 85% of breast cancers are HER2 negative [61].	100.0%

Variable in *PREDICT v3*	Mapping to MA.27	Missingness
Ki-67 Status	Information on Ki-67 was not available in MA.27 but missingness could be internally handled by *PREDICT v3*.	100.0%
Tumor Size in mm	Information on tumor size in mm was available for most patients. This information could not be inferred using background information but is required by *PREDICT v3* such that survival could not be estimated for these patients using *PREDICT v3*.	20.7%
Tumor Grade	This reflects the pathological tumor grading. MA.27 used a grading from 1 (well-differentiated) to 3 (poorly differentiated). This information could not be inferred using background information but is required by *PREDICT v3* so that survival could not be estimated for these patients using *PREDICT v3*.	21.9%
Mode of Detection	This variable was not available in the MA.27 dataset. In line with recent *PREDICT v3* validation studies [44], however, we added the following imputation process based on age and screening practices to *PREDICT v3*. For those within the standard screening programme age range (between 50-75 years), we set the mode of detection value to 0.5 to account for the proportion of eligible women who have regular screening in this population and the proportion of cancers in women who are interval cancers in those who have regular screening. For those outside the screening programme age range, mode of detection was set as symptomatic.	100.0%
Number of Nodes	*PREDICT v3* requires the exact number of nodes involved. MA.27 provided lymph node information through the TNM classification (see limitations in the main manuscript) so that we used the following approximations: N0 as 0 nodes, N1 as 2 nodes, N2 as 7 nodes and N3 as 10 nodes. Survival could not be estimated for patients with missing TNM classification using *PREDICT v3*.	0.1%
Micrometastases	Information on micrometastases is relevant in case of one positive node which would set the value of node to 0.5 instead of 1. This was not relevant since the TNM-based approximation for the number of nodes did not yield a value of exactly one positive node. Therefore, information on micrometastases is not applicable in this context.	100.0%
Radiotherapy	The MA.27 dataset did provide details on radiotherapy. Survival could not be estimated for patients with missing information on radiotherapy using *PREDICT v3.*	28.9%
Mean Heart Dose for Radiotherapy	In case of radiotherapy, the mean heart dose is required for *PREDICT v3*. This was not available in MA.27 but *PREDICT v3* provides the following instruction: "If you are unsure, use 0 for cancer on the right-hand side and 2 for cancer on the left-hand side." MA.27 provided tumor laterality for all patients who underwent radiotherapy so that we followed these instructions.	100.0%
Hormone Therapy	All patients in MA.27 received hormone therapy (see clinical trial details [59]).	0.0%

Variable in *PREDICT v3*	Mapping to MA.27	Missingness
Chemotherapy	While MA.27 provided complete information whether or not chemotherapy was part of the treatment, details regarding whether it was standard anthracycline or taxanes / high-dose anthracycline are missing. Taxanes (paclitaxel and docetaxel) have been introduced as chemotherapy for metastatic breast cancer in 1994 and 1996 by the FDA [62]. However, there was still an on-going debate on their use in the adjuvant setting [63,64] at the time of MA.27. A Canadian Medical Guideline on adjuvant systemic therapy for women with node-negative breast cancer around that time (2001 update) concluded that taxanes (more precisely AC-Taxol) had not been evaluated in node negative disease at that time. Similarly, the Canadian guideline on adjuvant systemic therapy for women with node-positive breast cancer (2001 update) mentioned that this is a field of research and that participation in trials investigating taxanes should be encouraged. MA21 was initiated by the National Cancer Institute of Canada-ClinicalTrials Group in 2000 based on evidence pointing towards a benefit of taxanes [65]. The rationale for the study [65] highlights that based on results of the CALGB 9344 study, AT/T can be considered as standard therapy for node-positive patients. Consequently, in MA21 the two standard regimens they compared against were CEF (Cyclophosphamid, Epirubicin and 5-Fluorouracil) and AC/T (doxorubicin and cyclophosphamide, followed by paclitaxel) whereby the first one can be considered as standard-dose anthracycline-based therapy and the second one as taxane-based therapy. The regimen they were interested in was EC/T (epirubicin and cyclophosphamide, followed by paclitaxel) which is a high-dose anthracycline and taxane-based therapy, and demonstrated superiority over AC/T but no superiority or inferiority over CEF [66]. Based on the context surrounding MA21 and insights from our co-authors (MC) who has been practicing in this field for decades, it can be concluded that taxanes/high-dose anthracycline-based therapy can be assumed for patients at the time of MA.27.	0.0%
Trastuzumab Therapy	The MA.27 dataset introduced trastuzumab usage as variable in 2005, following the publication of positive results showing efficacy of trastuzumab in HER2-positive early breast cancer patients. As a result, the majority of patients had no information on trastuzumab usage, most of whom were likely enrolled prior to 2005, when trastuzumab was not yet standard of care. Given this historical context, we assumed no trastuzumab use in cases with missing data.	74.7%
Bisphosphonate Use	The original publication mentions concurrent bisphosphonate use in 809/7,576 (11%), but this variable was absent in the MA.27 data we possess. Since bisphosphonate in non-metastatic breast cancer is rather unusual and not standard treatment, we assumed no bisphosphonate therapy.	100.0%

Table 2. Mapping MA.27 Variables to *PREDICT v3*. Missingness is indicated in the original variables before imputing as described in the table. The total number of individuals from MA.27 was 7,563.

**Variable in *PREDICT v3***	**Mapping to SEER**	**Missingness**
Year of Diagnosis	Year of diagnosis was known in SEER and those patients that had the same year of diagnosis as MA.27 were selected (i.e., 2003)	0.0%
Age in Years	Age in years was available for all patients.	0.0%
Postmenopausal Status	Postmenopausal status was not available in SEER but could be approximated by the age of the patients. An age below 45 years was considered as premenopausal.	100.0%
Smoking Status	As smoking status is required by *PREDICT v3* but was not available in SEER, we assumed smoker status as 0 (i.e., non-smoker). The same limitation applies as for the MA.27 data in this regard.	100.0%
Estrogen Receptor Status	Information on estrogen receptor status was available for all patients in our SEER subset.	0.0%
Progesterone Receptor Status	Information on progesterone receptor status was not available for all patients. This information is not necessarily required by *PREDICT v3* but can be left as missing.	3.8%
HER2 Status	SEER did not provide details on the HER2 status. We assumed HER2 negativity in line with the assumptions in the context of MA.27.	100.0%
Ki-67 Status	Information on Ki-67 was not available in SEER but missingness could be internally handled by *PREDICT v3*.	100.0%
Tumor Size in mm	Information on tumor size in mm was available for most patients. Survival could not be estimated for patients with missing information on tumor size using *PREDICT v3*.	6.1%
Tumor Grade	This reflects the pathological tumor grading. SEER used a grading from 1 (well-differentiated) to 4 (undifferentiated, anaplastic). We mapped grade 4 to grade 3, reflecting their shared classification as poorly differentiated tumors. Survival could not be estimated for patients with missing information on tumor grade using *PREDICT v3*.	7.2%
Mode of Detection	This variable was not available in the SEER data. We inferred mode of detection as done for MA.27.	100.0%
Number of Nodes	*PREDICT v3* requires the exact number of nodes involved. In contrast to MA.27, this information is available for most patients in SEER. Survival could not be estimated for patients with missing information on nodes using *PREDICT v3*.	10.2%
Micrometastases	Information on micrometastases is relevant in case of one positive node which would set the value of node to 0.5 instead of 1. We assumed that no micrometastases were present in the case of 1 positive node. This assumption affected 12.2% of all patients.	100.0%
Radiotherapy	The SEER dataset did provide details on radiotherapy. We aggregated any form of radiotherapy such as beam radiation, radioactive implants, radioisotopes or no specified method under radiotherapy.	0.0%
Mean Heart Dose for Radiotherapy	In case of radiotherapy, the mean heart dose is required for *PREDICT v3*. We inferred mode of detection as done for MA.27, but tumor laterality was missing in 0.01% where we assumed 1 Gray.	100.0%

Variable in ***PREDICT v3***	**Mapping to SEER**	**Missingness**
Hormone Therapy	Hormone therapy was not directly available in SEER, but it could be assumed for all hormone receptor positive patients.	100.0%
Chemotherapy	While SEER similar to MA.27 provided information whether or not chemotherapy was part of the treatment, details regarding whether it was standard anthracycline or taxanes / high-dose anthracycline are missing. We made the same assumptions as for MA.27.	100.0%
Trastuzumab Therapy	SEER did not provide details on trastuzumab therapy nor on HER2. Since we assumed HER2 negativity, we also assumed no trastuzumab therapy.	100.0%
Bisphosphonate Use	SEER did not provide information on bisphosphonate use but based on the same considerations as MA.27, we assumed no bisphosphonate therapy.	100.0%

Table 3. Mapping SEER Variables to *PREDICT v3*. The total number of individuals from SEER was 27,064.

Variable in ***PREDICT v3***	**Mapping to TEAM**	**Missingness**
Year of Diagnosis	Patient recruitment in TEAM covered a period from 2000 to 2006. Again, year of diagnosis can be assumed to be close to enrollment so that, similar to MA.27, the year of diagnosis was set to 2003 in all cases.	100%
Age in Years	Age in years was available for all patients.	0.00%
Postmenopausal Status	All patients were postmenopausal in TEAM (see eligibility criteria in [67])	0.00%
Smoking Status	As smoking status is required by *PREDICT v3* but was not available in TEAM, we assumed smoker status as 0 (i.e., non-smoker). The same limitation applies as for the MA.27 data in this regard.	100%
Estrogen Receptor Status	Information on estrogen receptor status was available for all but one patient in TEAM. Survival could not be estimated for this patient.	0.03%
Progesterone Receptor Status	Information on progesterone receptor status was not available for all patients. This information is not necessarily required by *PREDICT v3* but can be left as missing.	12.9%
HER2 Status	Similar to MA.27, the HER2 status was not available for all participants of the TEAM trial. In line with MA.27, we decided to assume HER2 negativity for patients where information about HER2 was not indicated (see limitations in main manuscript). This may better reflect clinical reality since around 85% of breast cancers are HER2 negative [61].	6.6%
Ki-67 Status	Information on Ki-67 was available in TEAM as percentage and was mapped to positive in case of > 10%. Missingness could be internally handled by *PREDICT v3*.	3.2%

Variable in *PREDICT v3*	Mapping to TEAM	Missingness
Tumor Size in mm	Information on tumor size in mm was available for most patients. Survival could not be estimated for patients with missing information on tumor size using *PREDICT v3*.	1.6%
Tumor Grade	This reflects the pathological tumor grading. Survival could not be estimated for patients with missing information on tumor grade using *PREDICT v3*.	4.8%
Mode of Detection	This variable was not available in the TEAM data. We inferred mode of detection as done for MA.27.	100%
Number of Nodes	*PREDICT v3* requires the exact number of nodes involved. In contrast to MA.27, this information is available for most patients in TEAM. Survival could not be estimated for patients with missing information on nodes using *PREDICT v3*.	16.1%
Micrometastases	Information on micrometastases is relevant in case of one positive node which would set the value of node to 0.5 instead of 1. We assumed that no micrometastases were present in the case of 1 positive node. This assumption affected 25.6% of all patients.	100%
Radiotherapy	The TEAM data did provide information on whether radiotherapy was applied. Survival could not be estimated for patients with missing information on radiotherapy using *PREDICT v3*.	0.2%
Mean Heart Dose for Radiotherapy	In case of radiotherapy, the mean heart dose is required for *PREDICT v3*. This was not available in TEAM nor information on tumor laterality to follow the instructions of *PREDICT v3* as mentioned above. Following the SEER procedure, we set the dose to 1 Gray in the case of radiotherapy which affected 64.9% of the patients.	100%
Hormone Therapy	All patients in TEAM received hormone therapy (see clinical trial details [67]).	0.0%
Chemotherapy	TEAM provided information on whether or not chemotherapy was part of the treatment, but did not provide consistent information about the regimen. To align with the trained models on MA.27, we set all chemotherapy applications to standard anthracyclines.	0.1%
Trastuzumab Therapy	TEAM did not provide details on trastuzumab therapy. We assumed no trastuzumab therapy in case of HER2 negativity, and trastuzumab therapy in case of positivity.	100%
Bisphosphonate Use	TEAM did not provide information on bisphosphonate use but based on the same considerations as MA.27, we assumed no bisphosphonate therapy.	100%

Table 4. Mapping TEAM Variables to *PREDICT v3*. The total number of individuals from TEAM was 3,825.

2.5 Stacked Ensemble

We implemented a stacked ensemble in which predictions from three individual models were combined*: f-PREDICT v3*, RSF and XGB using a constrained linear meta-learner. The models' predictions were linearly stacked, with non-negative weights constrained to sum to one (i.e., a convex combination). The weights were treated as continuous hyperparameters and the best combination was searched via Bayesian optimization. Bayesian optimization was implemented via the R package rBayesianOptimization [68]. Since *f-PREDICT v3* cannot generate survival estimates when certain inputs are missing, the ensemble was designed to fall back to the remaining models, RSF and XGB, in such cases. As a result, the ensemble could still provide robust predictions even when there was incomplete input data.

3. Supplemental Results

3.1 Descriptive Characteristics of MA.27

Model training was conducted on MA.27. We present descriptive visualizations of the cohort in addition to the summary in the main manuscript. **Figure 1** shows the Kaplan-Meier estimate of 5-year breast cancer specific survival. It illustrates the extent of right-censoring within the follow-up period. **Figure 2** and **Figure 3** show the distribution of relevant clinicopathological variables stratified by 5-year event status.

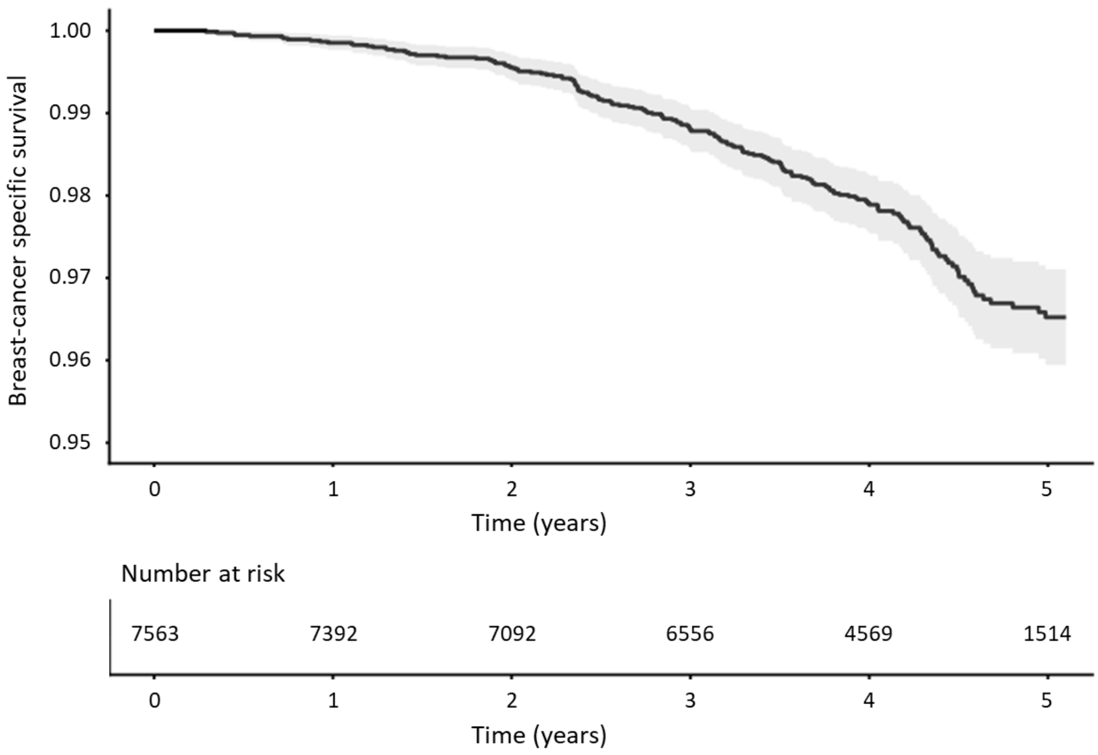

Figure 1. Kaplan-Meier Estimate for 5-Year Breast-Cancer Specific Survival in MA.27. The curve indicates the cumulative survival probability within the follow-up time. The number at risk is shown below the x-axis and refers to the individuals who are still under follow-up and have not yet experienced the event just prior to the indicated time.

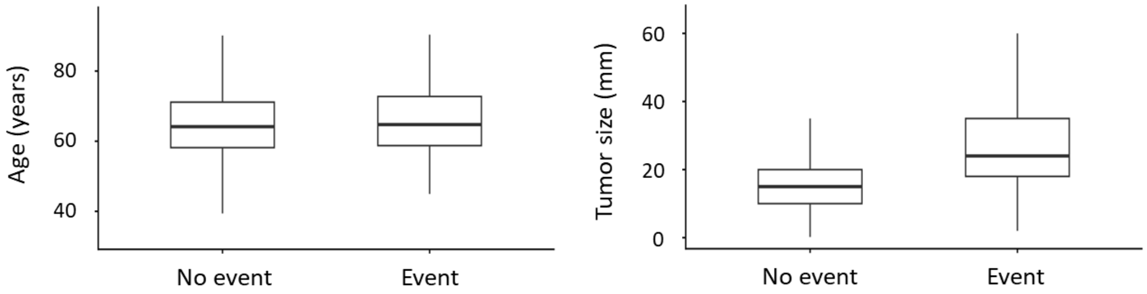

Figure 2. Distribution of Age and Tumor Size Stratified by 5-Year Event Status in MA.27. Boxplots illustrate the distribution among individual with and without breast cancer related death within 5 years.

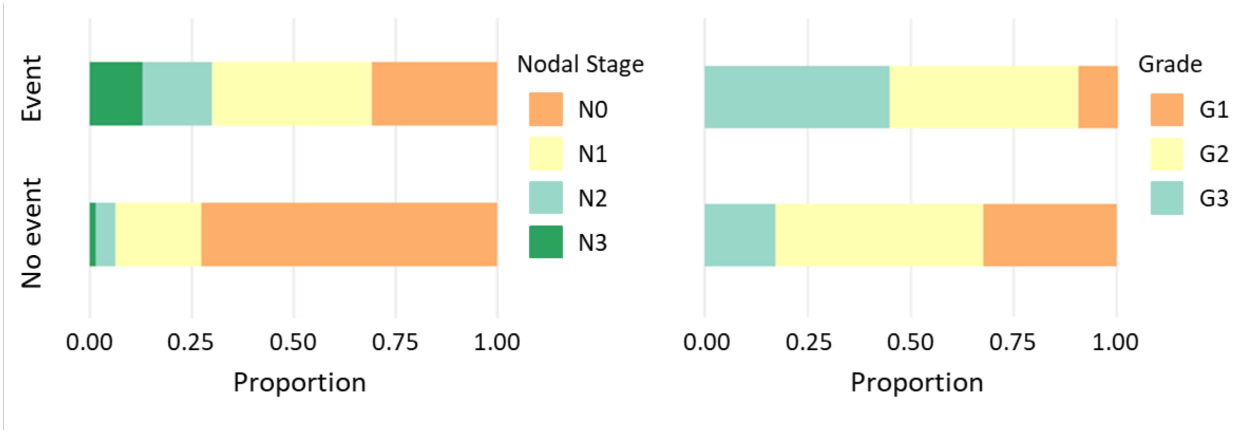

Figure 3. Nodal Stage and Tumor Grade Stratified by 5-Year Event Status in MA.27. Horizontal stacked bar plots show the percentage distribution of the nodal stages and the grades within each group.

3.2 Missing Data Patterns in MA.27

Variable-level availability and assumptions for *PREDICT v3* inputs are summarized in **Table 2**, **Table 3** and **Table 4** of this appendix for all three cohorts, MA.27, SEER and TEAM, respectively. As discussed, several variables were structurally absent and were therefore handled through explicit, clinically grounded assumptions. Sensitivity analyses assessing the impact of these assumptions are presented in **Appendix A: Supplemental Results – Sensitivity Analysis With Best and Worse Case Assumptions**.

The following analyses characterize missingness in variables that were part of MA.27. Their missingness pattern is detailed in **Figure 4.** The median number of missing variables per record was 1 (IQR 1-1.5), indicating that most patients had incomplete information for at least one variable. This variable was most often trastuzumab (see **Figure 4**) which was introduced later during the study period (in 2008) and therefore not consistently collected.

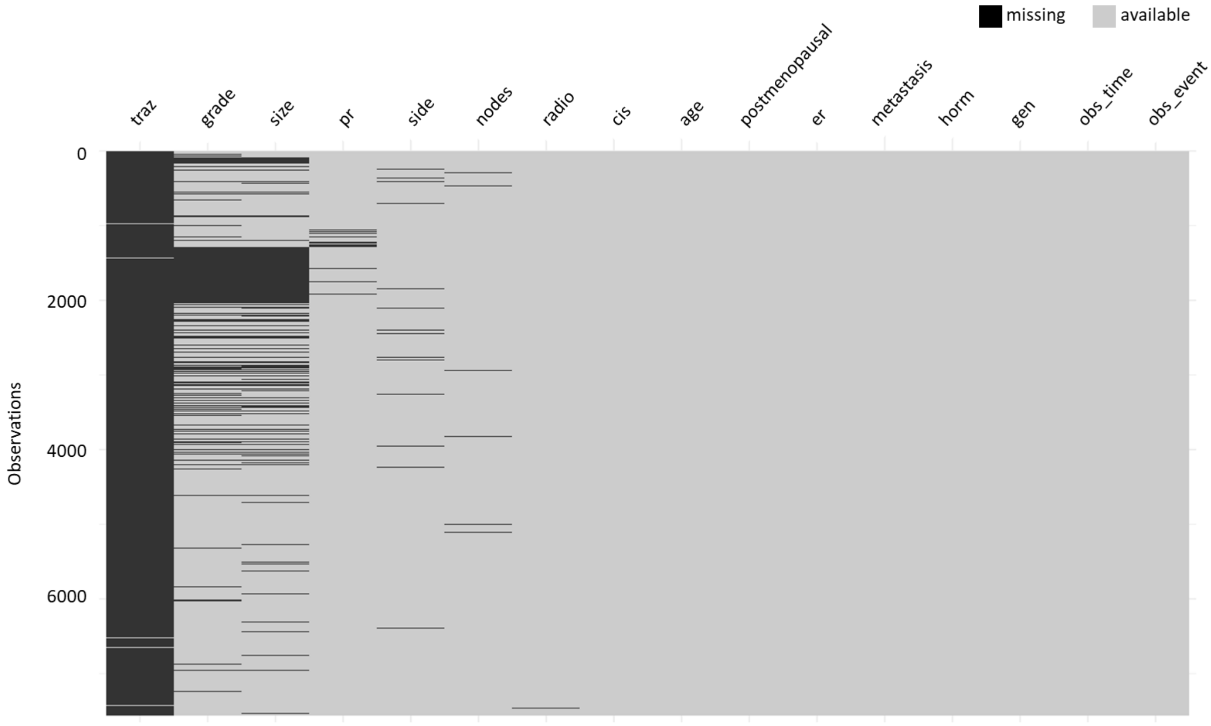

Figure 4. Missingness Pattern in MA.27. age: age in years; cis: women with DCIS/LCIS only; er: estrogen receptor status; gen: chemotherapy; grade: pathological grading; horm: hormone therapy; metastasis: women with metastatic disease; nodes: number of positive nodes; obs_time: time-to-event or follow-up time; obs_event: observed event; postmenopausal: postmenopausal status; pr: progesterone receptor status; radio: radiotherapy; side: tumor laterality; size: size of tumor; traz: trastuzumab therapy.

We further assessed whether missingness was informative with respect to survival outcomes through univariable Cox proportional hazards models. Missingness was thereby coded as a binary indicator for each variable with non-zero missingness (i.e., traz, grade, size, pr, side, nodes, radio).

Across these variables, missingness indicators showed no associations with survival outcomes (see **Table 5**) with some extreme hazard ratio (HR) estimates and/or 95% CI, reflecting sparse event counts and unbalanced missing and non-missing groups.

Variable	HR	95% CI
traz	1217427.12	0-Inf
grade	0.99	0.68-1.45
size	1.11	0.76-1.62
pr	0.24	0.03-1.73
side	2.98	0-Inf
nodes	0.42	0.06-3.02
radio	6.14	0-Inf

Table 5. Cox Proportional Hazards Models with Missingness Indicators. grade: pathological grading; nodes: number of positive nodes; pr: progesterone receptor status; radio: radiotherapy; side: tumor laterality; size: size of tumor; traz: trastuzumab therapy

3.3 Model Performance Using Imputed Data

Data was imputed as described earlier and results from the internal validation are shown in **Table 6**. As in the main manuscript, the models were optimized for ICI during training. Discrimination and calibration did not considerably change with imputation.

Model	Calibration (ICI)		Discrimination (AUROC)	
	**Median**	**IQR**	**Median**	**IQR**
**Imputed Dataset**				
PREDICT v3	0.042	0.039-0.044	0.738	0.719-0.770
f-PREDICT v3	0.006	0.003-0.100	0.800	0.789-0.827
RSF	0.003	0.003-0.008	0.745	0.743-0.755
XGB	0.039	0.034-0.041	0.773	0.744-0.800
Ensemble	0.008	0.006-0.022	0.747	0.726-0.765
**Non-Imputed Dataset**				
PREDICT v3	0.042	0.039-0.047	0.738	0.719-0.719
f-PREDICT v3	0.005	0.004-0.010	0.799	0.789-0.818
RSF	0.003	0.002-0.008	0.744	0.731-0.760
XGB	0.040	0.038-0.043	0.783	0.764-0.810
Ensemble	0.007	0.003-0.009	0.746	0.733-0.766

Table 6. Discrimination (AUROC) and Calibration (ICI) When Testing Imputation. AUROC and ICI values were calculated on the validation dataset and the median with IQR over 10 seed settings is indicated per model. Training was done on the imputed, non-rebalanced dataset. Results from the non-imputed, non-rebalanced dataset used in the main analysis are also shown for reference. Training was optimized for ICI.

3.4 Model Performance Under Best and Worse Case Assumptions

Sensitivity analyses using optimistic and pessimistic clinical assumptions revealed different variability in the performance across models. While the pre-trained *PREDICT v3* model showed the strongest sensitivity to input assumptions, particularly in terms of calibration, *f-PREDICT v3* was less sensitive, and the de-novo ML models and the stacked ensemble demonstrated stable calibration and discrimination across all scenarios (see **Table 7**).

Model	Calibration (ICI)		Discrimination (AUROC)	
	**Median**	**IQR**	**Median**	**IQR**
**Best Case**				
PREDICT v3	0.027	0.024-0.029	0.710	0.705-0.745
f-PREDICT v3	0.010	0.007-0.014	0.743	0.724-0.774
RSF	0.007	0.002-0.008	0.744	0.731-0.760
XGB	0.040	0.038-0.043	0.783	0.764-0.810
Ensemble	0.007	0.004-0.007	0.740	0.730-0.763
**Worse Case**				
PREDICT v3	0.073	0.070-0.078	0.768	0.743-0.798
f-PREDICT v3	0.013	0.012-0.017	0.821	0.818-0.848
RSF	0.005	0.002-0.007	0.744	0.725-0.764
XGB	0.040	0.037-0.043	0.796	0.764-0.810
Ensemble	0.007	0.004-0.007	0.740	0.724-0.763
**Clinically Most Plausible Case**				
PREDICT v3	0.042	0.039-0.047	0.738	0.719-0.719
f-PREDICT v3	0.005	0.004-0.010	0.799	0.789-0.818
RSF	0.003	0.002-0.008	0.744	0.731-0.760
XGB	0.040	0.038-0.043	0.783	0.764-0.810
Ensemble	0.007	0.003-0.009	0.746	0.733-0.766

Table 7. Discrimination (AUROC) and Calibration (ICI) Under Best and Worse Case Assumptions. AUROC and ICI values were calculated on the validation dataset and the median with IQR over 10 seed settings is indicated per model. Training was done on the non-rebalanced and non-imputed dataset. Results from the clinically most plausible case used in the main analysis are shown for reference. Training was optimized for ICI.

3.5 Re-Balancing For Model Training

As in the main manuscript, the models were optimized for ICI and re-balancing at the dataset level and at the algorithm level were tested. Results of training with re-balancing in **Table 8** show that calibration deteriorated when leveraging ROSE during training.

At the algorithm level, a target weighted event fraction of 0.20 was selected by majority vote for both RSF and XGB during hyperparameter tuning. Despite this substantial upweighting, algorithm-level re-balancing did not improve performance, but deterioration was less severe than in dataset-level re-balancing. The stacked ensemble had a comparatively higher stability than the individual ML models. However, this is very likely attributable to the contribution of the well-calibrated *f-PREDICT v3* within the ensemble that was not affected by re-balancing on the algorithm-level.

**Model**	**Calibration (ICI)**		**Discrimination (AUROC)**	
	**Median**	**IQR**	**Median**	**IQR**
**Re-balancing at the dataset level**				
f-PREDICT v3	0.334	0.328-0.335	0.812	0.778-0.838
RSF	0.247	0.232-0.266	0.784	0.764-0.798
XGB	0.554	0.549-0.565	0.792	0.786-0.802
Ensemble	0.287	0.238-0.318	0.787	0.768-0.807
**Re-balancing at the algorithm level**				
RSF	0.017	0.013-0.018	0.727	0.705-0.748
XGB	0.083	0.067-0.965	0.664	0.534-0.696
Ensemble	0.015	0.011-0.037	0.717	0.679-0.748
**No re-balancing**				
PREDICT v3	0.042	0.039-0.047	0.738	0.719-0.719
f-PREDICT v3	0.005	0.004-0.010	0.799	0.789-0.818
RSF	0.003	0.002-0.008	0.744	0.731-0.760
XGB	0.040	0.038-0.043	0.783	0.764-0.810
Ensemble	0.007	0.003-0.009	0.746	0.733-0.766

Table 8. Discrimination (AUROC) and Calibration (ICI) When Testing Re-Balancing. AUROC and ICI values were calculated on the validation dataset and the median with IQR over 10 seed settings is indicated per model. Re-balancing was implemented either (i) at the dataset level using ROSE applied to the training data prior to ML model training and *PREDICT v3* fine-tuning, or (ii) via case weights applied during ML training. Only models affected by re-balancing are shown in the respective table sections. Results from all models trained on the non-imputed and non-rebalanced datasets are shown for reference. Training was optimized for ICI.

3.6 AUROC Optimization for Model Training

We also used the AUROC as the optimization goal during training. While discrimination is a relevant ability for models in general, it is less relevant for decision making in prognostication tools.

The discriminative performance (i.e., AUROC) for AUROC-optimized models is shown in **Table 9** when trained with and without re-balancing. Again, as with ICI-optimized models, re-balancing via ROSE (i.e., at the dataset level) did decrease calibration and had varying small effects on discrimination. Re-balancing at the algorithm level likewise did not improve performance. The negative impact, however, was less pronounced than under dataset-level re-balancing.

**Model**	**Calibration (ICI)**		**Discrimination (AUROC)**	
	**Median**	**IQR**	**Median**	**IQR**
**No re-balancing**				
PREDICT v3	0.042	0.039-0.049	0.738	0.719-0.770
f-PREDICT v3	0.103	0.057-0.127	0.803	0.803-0.844
RSF	0.008	0.003-0.011	0.757	0.732-0.797
XGB	0.044	0.039-0.047	0.779	0.747-0.820
Ensemble	0.043	0.037-0.045	0.795	0.787-0.813
**Re-balancing at the dataset level**				
f-PREDICT v3	0.172	0.143-0.242	0.828	0.797-0.842
RSF	0.274	0.262-0.322	0.795	0.775-0.810
XGB	0.563	0.551-0.568	0.799	0.766-0.811
Ensemble	0.469	0.434-0.496	0.800	0.771-0.813
**Re-balancing at the algorithm level**				
RSF	0.020	0.019-0.029	0.743	0.710-0.788
XGB	0.214	0.167-0.267	0.763	0.750-0.792
Ensemble	0.159	0.067-0.213	0.798	0.749-0.825

Table 9. Discrimination (AUROC) and Calibration (ICI) When Optimizing for AUROC. AUROC and ICI values were calculated on the validation dataset and the median with IQR over 10 seed settings is indicated per model. Training was done without and with re-balancing. Re-balancing was implemented either (i) at the dataset level using ROSE applied to the training data prior to ML model training and *PREDICT v3* fine-tuning, or (ii) via case weights applied during ML training. Only models affected by re-balancing are shown. Training was optimized for AUROC.

3.7 External Evaluation Plots

External evaluation was conducted on data from the US SEER program and on the clinical trial dataset TEAM. ICI and AUROC values are presented in the main manuscript. **Figure 5** and **Figure 6** provide the graphical illustration of calibration and discrimination for SEER; **Figure 7** and **Figure 8** for TEAM. The illustrations confirm the mixed findings from the main manuscript: while *PREDICT v3* performed similar across all datasets, MA.27 tailored transfer learning, de-novo ML and the stacked ensemble did outperform the pre-trained models in terms of discrimination and calibration in SEER but not in TEAM. In TEAM, all models consistently overestimated survival.

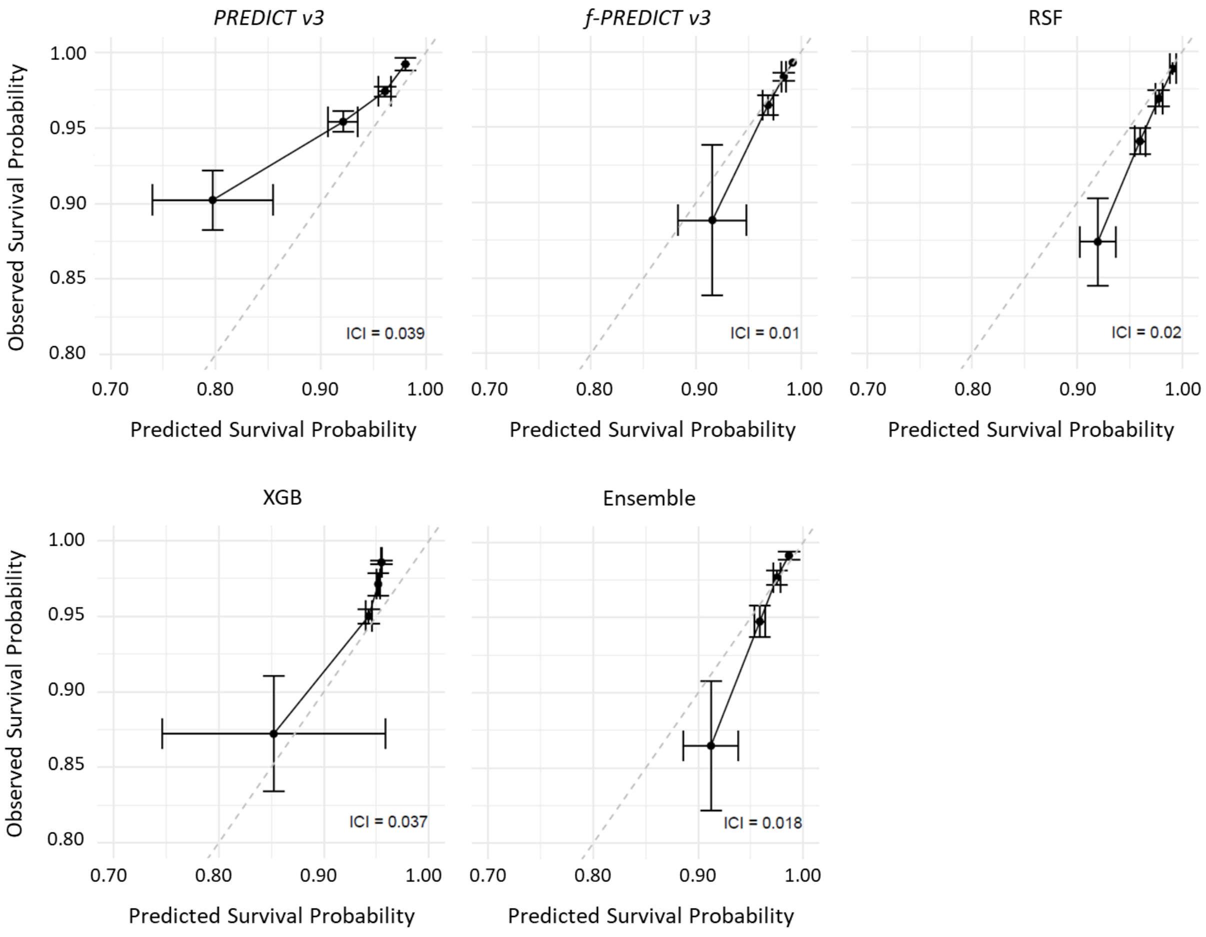

Figure 5. Calibration Plots for External Evaluation on SEER. Calibration plots are illustrated for the baseline model *PREDICT v3* as well as the four models enhanced by transfer learning (i.e., *f-PREDICT v3*), ML (RSF and XGB) and the stacked ensemble. The diagonal dashed line indicates perfect calibration. Observed probabilities were smoothed using a hazard regression-based method [69]. Observations were then divided into four quartiles based on their predicted probabilities. Both predicted and observed probabilities were trimmed to exclude extreme values beyond the 10th to 90th percentile, and mean predicted and observed survival probabilities were calculated in each quartile. The horizontal and vertical error bars reflect the standard deviations of these quartile-wise means. The calculated ICI is given for each model.

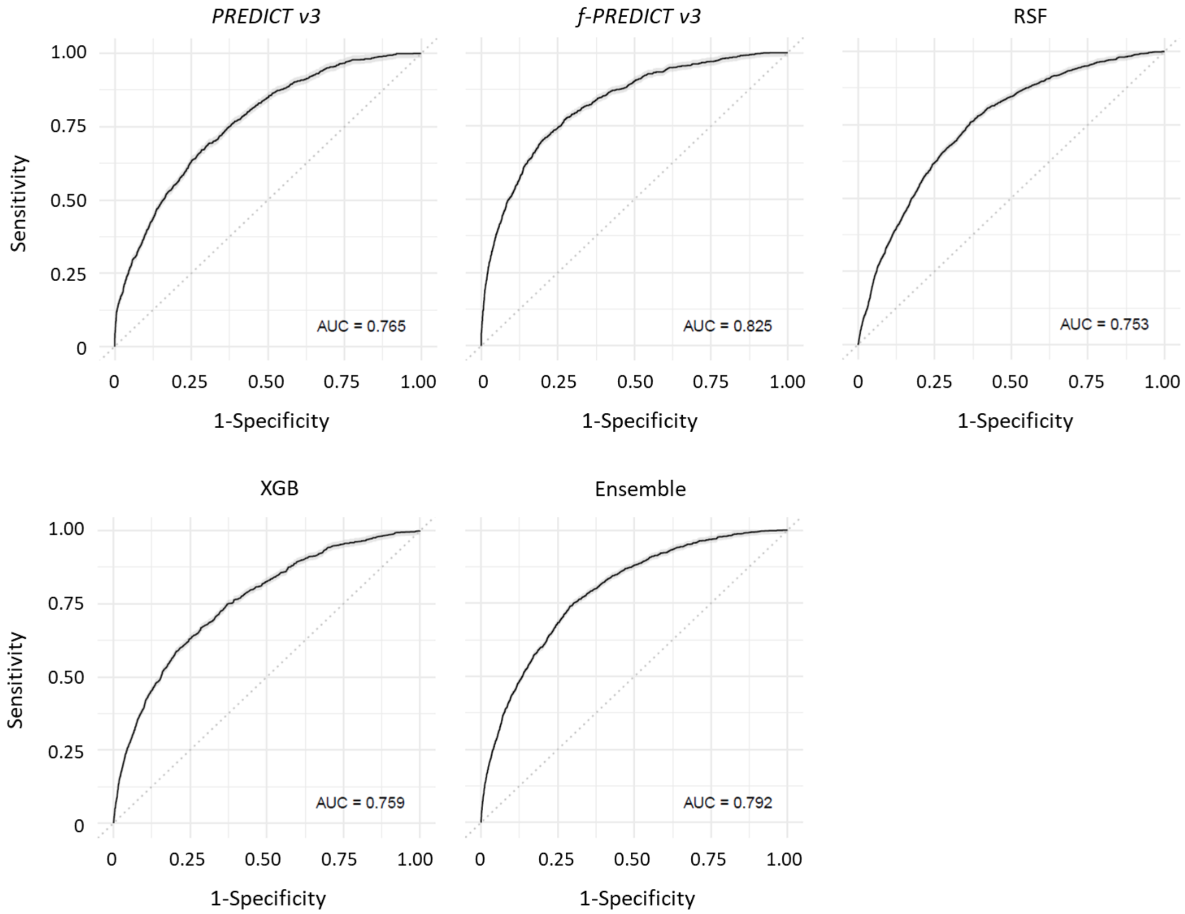

Figure 6. Receiver Operating Characteristics (ROC) Curves for SEER. ROC curves are illustrated for the baseline model *PREDICT v3* as well as the four models enhanced by transfer learning (i.e., *f-PREDICT v3*), ML (RSF and XGB) and the stacked ensemble. The diagonal dashed grey line indicates discrimination of a random guess. The calculated AUROC value is given for each model.

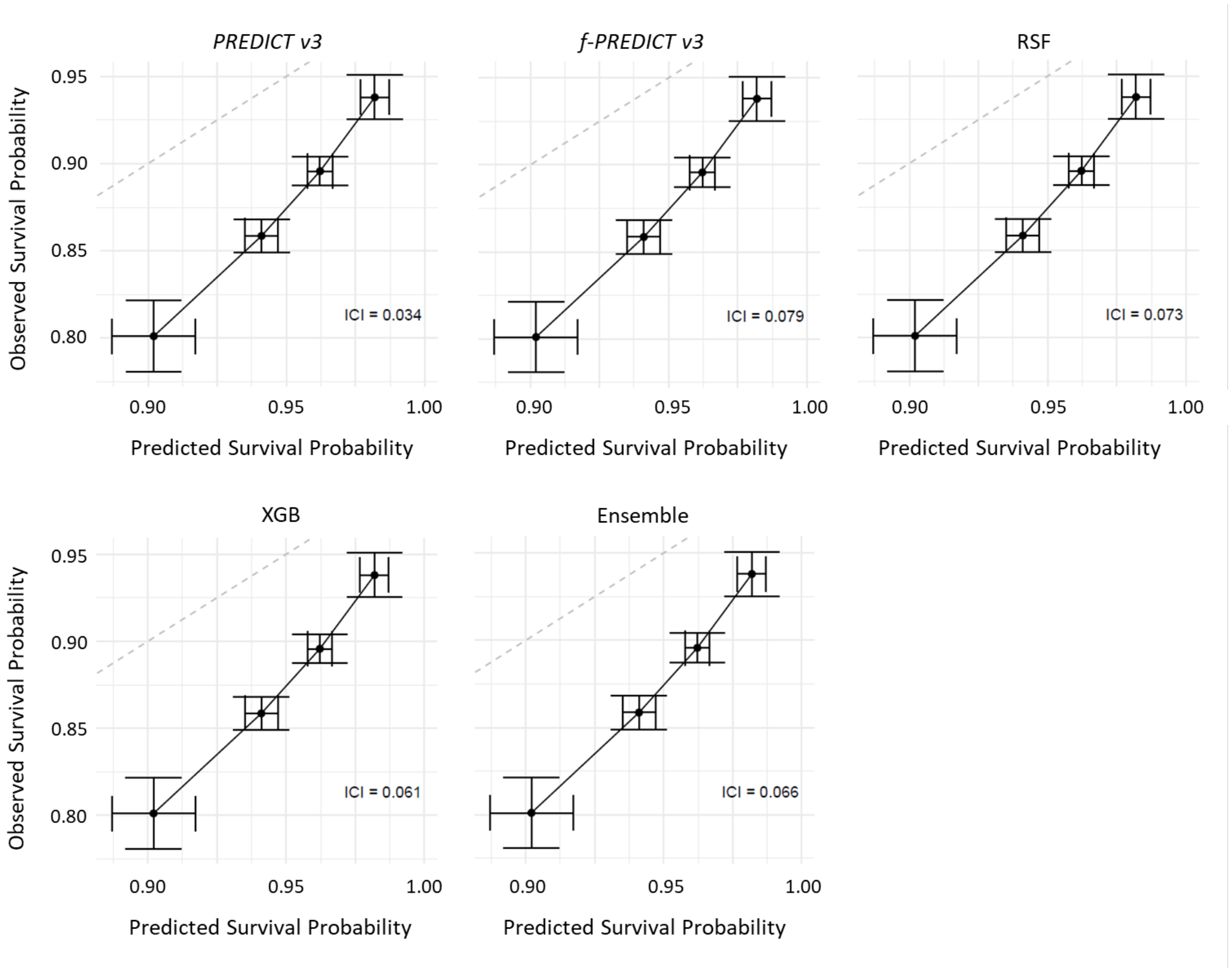

Figure 7. Calibration Plots for External Evaluation on TEAM. Calibration plots are illustrated for the baseline model *PREDICT v3* as well as the four models enhanced by transfer learning (i.e., *f-PREDICT v3*), ML (RSF and XGB) and the stacked ensemble. The diagonal dashed line indicates perfect calibration. Observed probabilities were smoothed using a hazard regression-based method [69]. Observations were then divided into four quartiles based on their predicted probabilities. Both predicted and observed probabilities were trimmed to exclude extreme values beyond the 10th to 90th percentile, and mean predicted and observed survival probabilities were calculated in each quartile. The horizontal and vertical error bars reflect the standard deviations of these quartile-wise means. The calculated ICI is given for each model.

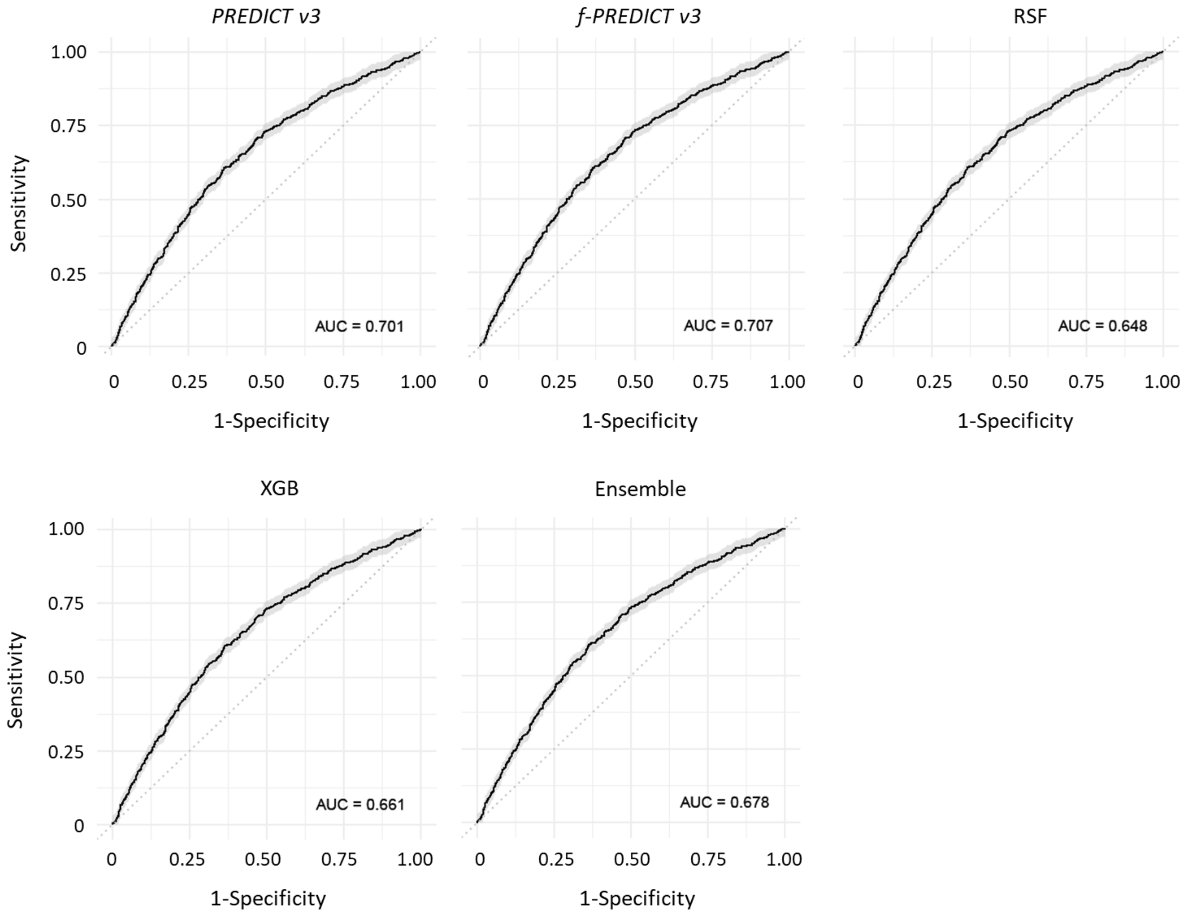

Figure 8. Receiver Operating Characteristics (ROC) Curves for TEAM. ROC curves are illustrated for the baseline model *PREDICT v3* as well as the four models enhanced by transfer learning (i.e., *f-PREDICT v3*), ML (RSF and XGB) and stacked ensemble. The diagonal dashed grey line indicates discrimination of a random guess. The calculated AUROC value is given for each model.

3.8 Stratified Results from External Validation on SEER

External evaluation was conducted on data from the US SEER program and on the clinical trial dataset TEAM. While performance on SEER was comparable to the internal validation, discrimination and calibration were reduced in TEAM. To better understand this discrepancy, stratified analyses were performed within the SEER cohort.

The strata were defined by nodal stage, tumor size and tumor grade. More precisely, a high-risk stratum was defined with characteristics (node positive, tumor size > 2cm, G3) that were more prevalent in the TEAM cohort. Within this stratum in SEER, model performance decreased substantially, with calibration and discrimination comparable to, and, in some cases (i.e., RSF, XGB), worse than those observed in TEAM. Similar to TEAM, survival was underestimated by the models in this stratum.

Results from the stratified analysis are shown in **Table 10**

Model	Calibration		Discrimination	
	ICI	95% CI	AUROC	95% CI
**Entire SEER Cohort (n=27,064)**				
PREDICT v3	0.039	0.037-0.198	0.765	0.750-0.780
f-PREDICT v3	0.010	0.009-0.173	0.825	0.811-0.838
RSF	0.020	0.019-0.046	0.753	0.741-0.765
XGB	0.037	0.034-0.093	0.759	0.747-0.771
Ensemble	0.018	0.016-0.078	0.792	0.779-0.802
**Stratum Node Positive, Size > 2cm, G3 (n=1,503)**				
PREDICT v3	0.024	0.020-0.179	0.716	0.680-0.750
f-PREDICT v3	0.060	0.055-0.149	0.723	0.688-0.753
RSF	0.106	0.102-0.119	0.612	0.577-0.647
XGB	0.190	0.164-0.270	0.650	0.615-0.687
Ensemble	0.070	0.066-0.151	0.699	0.663-0.733
**Stratum Node Negative, Size ≤ 2cm, G1/G2 (n=20,229)**				
PREDICT v3	0.044	0.043-0.083	0.731	0.713-0.748
f-PREDICT v3	0.008	0.006-0.232	0.797	0.779-0.813
RSF	0.002	0.001-0.061	0.733	0.715-0.753
XGB	0.070	0.036-0.077	0.755	0.737-0.774
Ensemble	0.008	0.006-0.171	0.779	0.762-0.796
**Entire TEAM Cohort (n=3,825)**				
PREDICT v3	0.034	0.028-0.058	0.701	0.672-0.732
f-PREDICT v3	0.079	0.072-0.089	0.707	0.677-0.736
RSF	0.073	0.071-0.076	0.648	0.623-0.673
XGB	0.061	0.051-0.091	0.661	0.635-0.687
Ensemble	0.073	0.071-0.076	0.678	0.651-0.703

Table 10. Stratified Calibration and Discrimination for SEER Data. Values were calculated on the external validation data. Stratification was conducted according to nodal stage, tumor size and tumor grade. Stratified cohorts exclude individuals with missing information for the respective information. The value and its 95% CI as derived from bootstrapping is indicated for all survival models. Training was done on the non-imputed and non-rebalanced MA.27 dataset and optimized for ICI. The entire SEER and TEAM cohort are shown for reference.

3.9 Parameter Variability Across 50 Independent Runs

Model training, testing and validation on MA.27 was conducted across 10 independent runs to account for variability across different splits. The ML parameters were then chosen by majority vote, the fine-tuned values for the 26 parameters in *PREDICT v3* and the ensemble weights by averaging. **Table 11**, **Table 12** and **Table 13** summarize the variability of model parameters across a larger set of 50 independent runs to assess model stability and sensitivity to data splits. This was conducted under the final modeling configuration used in the main analyses, namely without re-balancing, without imputation, and with optimization for ICI.

Across these 50 independent runs, ML hyperparameters showed varying degrees of stability. For some parameters, such as the number of variables sampled at each split in RSF (mtry), the splitting criterion in RSF (splitrule) and the learning rate in XGB (eta), a single value clearly dominated across runs. For other parameters selection frequencies were more evenly distributed across the parameter values, indicating comparable performance across different parameterizations (see **Table 11**). The hyperparameter selection based on 10 independent runs was consistent with the dominant values observed across the 50 runs. Few hyperparameters selected based on 10 runs differed from the majority vote across 50 runs, namely the minimum terminal node size in RSF (nodesize), the subsample ratio of columns in XGB (colsample_bytree) and the L2 regularization coefficient in XGB (lambda_vals). These parameters were more evenly distributed in general, suggesting limited sensitivity to the exact parameter choice rather than sensitivity to the specific data split.

During transfer-learning, the parameters of *PREDICT v3* were fine-tuned to the MA.27 data. Across 50 independent runs, fine-tuning was limited to a small subset of parameters while the majority remained identical to the original *PREDICT v3* parameters (see **Table 12**). Within this subset, variability across runs was limited (maximum SD 1.31) and no contradictory behavior across splits was observed. More importantly, the values selected based on 10 runs were consistent with these patterns.

While the weights for the stacked ensemble showed variability across the 50 independent runs, an overall tendency toward prioritization of RSF could be observed, followed by *f-PREDICT v3* and then XGB. This tendency was more pronounced in the average across 50 runs compared with the average based on 10 runs.

	Hyperparameter	Value	Selected in Runs (n = 10)
RSF	ntree	**500**	19
		1000	17
		1500	14
	mtry	**3**	27
		4	10
		5	13
	nodesize	3	5
		5	12
		10	18
		**15**	15
	splitrule	logrank	3
		**logrankscore**	47
XGB	eta	**0.05**	39
		0.1	11
	max_depth	2	25
		**5**	25
	subsample	**0.6**	29
		1	21
	colsample_bytree	0.6	29
		**1**	21
	lambda_vals	**0.05**	22
		0.1	28

Table 11. ML Hyperparameters Across 50 Independent Runs. The final model parameters as selected based on 10 independent runs are printed in bold.

Parameter	Original Value	Mean	SD	Selected Value
ag_ot_ea.beta.1	4.21	6.66	1.31	7.00
ag_ot_ea.beta.2	-31.41	-31.22	0.75	-31.06
ag1_er0	1.76	1.82	0.44	1.76
ag1_er1	0.20	0.20	0.00	0.20
ag2_er0	4.56	4.56	0.00	4.56
ag2_er1	2.93	2.93	0.00	2.93
gr1_er0	0.35	0.35	0.00	0.35
gr1_er1	0.71	0.71	0.00	0.71
h_ot_i	-4.85	-4.85	0.00	-4.85
h_ot_t1	1.34	4.16	0.95	4.45
h_ot_t2	0.50	0.50	0.00	0.50
h0_br_i	-3.02	-3.02	0.00	-3.02
h0_br_t1	-0.58	-0.58	0.00	-0.58
h0_br_t2	-0.10	-0.04	0.44	-0.10
h1_br_i	-2.32	-2.32	0.00	-2.32
h1_br_t1	-3.62	-3.62	0.00	-3.62
h1_br_t2	-0.54	-0.29	0.86	-0.54
nd1_er0	0.63	0.63	0.00	0.63
nd1_er1	0.67	0.67	0.00	0.67
sc1_er0	-0.21	-0.09	0.62	-0.21
sc1_er1	-0.32	-0.32	0.00	-0.32
sz1_er0	0.74	0.74	0.00	0.74
sz1_er1	2.27	2.27	0.00	2.27
yr_ot_ea.beta	-0.02	-0.02	0.00	-0.02
yr1_er0	-0.05	-0.05	0.00	-0.05
yr1_er1	-0.05	0.14	0.75	-0.05

Table 12. *f-PREDICT v3* Parameters Across 50 Independent Runs. The selected parameter values are based on the mean across 10 independent runs. The original value is the one from *PREDICT v3* before transfer learning. SD: standard deviation.

		Model	Mean	SD	Selected Value
**Raw Weights**	**ST**	rsf	0.82	0.32	0.61
		xgb	0.39	0.38	0.22
	**FB**	rsf	0.73	0.31	0.80
**Implied Weights**	**ST**	rsf	0.82	0.32	0.61
		xgb	0.02	0.05	0.09
		f-PREDICT v3	0.16	0.32	0.30
	**FB**	rsf	0.73	0.31	0.80
		xgb	0.27	0.31	0.20

Table 13. Ensemble Weights Across 50 Independent Runs. Raw weights correspond to the optimized convex-combination parameters of the stacked ensemble. Implied weights represent the effective contributions of each model after applying the raw weights. Standard (ST) refers to observations for which *f-PREDICT v3* predictions are available; fallback (FB) to observations with missing *f-PREDICT v3* predictions where the ensemble is reduced to RSF and XGB. The selected weight values are based on the mean across 10 independent runs. SD: standard deviation.

4. References

1 Kaplan EL, Meier P. Nonparametric Estimation from Incomplete Observations. *Breakthroughs in Statistics*. Springer New York 1992:319–37.

2 Cox DR. Regression Models and Life-Tables. *Journal of the Royal Statistical Society Series B (Methodological)*. 1972;34:187–220.

3 Rodriguez G. Parametric survival models. *Int J Comput Algorithm*. 2010.

4 Hougaard P. Frailty models for survival data. *Lifetime Data Analysis*. 1995;1:255–73. doi: 10.1007/bf00985760

5 Wei LJ. The accelerated failure time model: A useful alternative to the cox regression model in survival analysis. *Statistics in Medicine*. 1992;11:1871–9. doi: 10.1002/sim.4780111409

6 Stroustrup N, Anthony WE, Nash ZM, *et al.* The temporal scaling of Caenorhabditis elegans ageing. *Nature*. 2016;530:103–7. doi: 10.1038/nature16550

7 Ishwaran H, Kogalur UB, Blackstone EH, *et al.* Random survival forests. *The Annals of Applied Statistics*. 2008;2. doi: 10.1214/08-aoas169

8 Chen T, Guestrin C. XGBoost: A Scalable Tree Boosting System. *Proceedings of the 22nd ACM SIGKDD International Conference on Knowledge Discovery and Data Mining*. San Francisco California USA: ACM 2016:785–94.

9 Hornik K. Approximation capabilities of multilayer feedforward networks. *Neural Networks*. 1991;4:251–7. doi: 10.1016/0893-6080(91)90009-t

10 Hornik K, Stinchcombe M, White H. Multilayer feedforward networks are universal approximators. *Neural Networks*. 1989;2:359–66. doi: 10.1016/0893-6080(89)90020-8

11 Katzman JL, Shaham U, Cloninger A, *et al.* DeepSurv: personalized treatment recommender system using a Cox proportional hazards deep neural network. *BMC Medical Research Methodology*. 2018;18:24. doi: 10.1186/s12874-018-0482-1

12 Lee C, Zame W, Yoon J, *et al.* DeepHit: A Deep Learning Approach to Survival Analysis With Competing Risks. *Proceedings of the AAAI Conference on Artificial Intelligence*. 2018;32. doi: 10.1609/aaai.v32i1.11842

13 Bennis A, Mouysset S, Serrurier M. DPWTE: A Deep Learning Approach to Survival Analysis Using a Parsimonious Mixture of Weibull Distributions. *Artificial Neural Networks and Machine Learning – ICANN 2021*. Springer International Publishing 2021:185–96.

14 Bice N, Kirby N, Bahr T, *et al.* Deep learning-based survival analysis for brain metastasis patients with the national cancer database. *Journal of Applied Clinical Medical Physics*. 2020;21:187–92. doi: 10.1002/acm2.12995

15 Faraggi D, Simon R. A neural network model for survival data. *Statistics in Medicine*. 1995;14:73–82. doi: 10.1002/sim.4780140108

16 Hu S, Fridgeirsson E, Wingen G van, *et al.* Transformer-Based Deep Survival Analysis. In: Greiner R, Kumar N, Gerds TA, *et al.*, eds. *Proceedings of AAAI Spring Symposium on Survival Prediction - Algorithms, Challenges, and Applications 2021*. PMLR 2021:132–48.

17 Mesinovic M, Watkinson P, Zhu T. DySurv: dynamic deep learning model for survival analysis with conditional variational inference. *Journal of the American Medical Informatics Association*. 2024;ocae271. doi: 10.1093/jamia/ocae271

18 Wiegrebe S, Kopper P, Sonabend R, *et al.* Deep learning for survival analysis: a review. *Artificial Intelligence Review*. 2024;57. doi: 10.1007/s10462-023-10681-3

19 Fouodo CJK, Knig IR, Weihs C, *et al.* Support Vector Machines for Survival Analysis with R. *The R Journal*. 2018;10:412–23.

20 Belle VV, Pelckmans K, Suykens JAK, *et al.* Survival SVM: a Practical Scalable Algorithm.

21 Ibrahim JG, Chen M-H, Sinha D. *Bayesian Survival Analysis*. New York, NY: Springer 2001.

22 Sparapani RA, Logan BR, McCulloch RE, *et al.* Nonparametric survival analysis using Bayesian Additive Regression Trees (BART). *Statistics in Medicine*. 2016;35:2741–53. doi: 10.1002/sim.6893

23 El Haji H, Souadka A, Patel BN, *et al.* Evolution of Breast Cancer Recurrence Risk Prediction: A Systematic Review of Statistical and Machine Learning–Based Models. *JCO Clin Cancer Inform*. 2023;e2300049. doi: 10.1200/CCI.23.00049

24 Li J, Zhou Z, Dong J, *et al.* Predicting breast cancer 5-year survival using machine learning: A systematic review. *PLOS ONE*. 2021;16:e0250370. doi: 10.1371/journal.pone.0250370

25 Huang Y, Li J, Li M, *et al.* Application of machine learning in predicting survival outcomes involving real-world data: a scoping review. *BMC Medical Research Methodology*. 2023;23. doi: 10.1186/s12874-023-02078-1

26 Moncada-Torres A, van Maaren MC, Hendriks MP, *et al.* Explainable machine learning can outperform Cox regression predictions and provide insights in breast cancer survival. *Sci Rep*. 2021;11:6968. doi: 10.1038/s41598-021-86327-7

27 Kurt Omurlu I, Ture M, Tokatli F. The comparisons of random survival forests and Cox regression analysis with simulation and an application related to breast cancer. *Expert Systems with Applications*. 2009;36:8582–8. doi: 10.1016/j.eswa.2008.10.023

28 Hamedi SZ, Emami H, Khayamzadeh M, *et al.* Application of machine learning in breast cancer survival prediction using a multimethod approach. *Sci Rep*. 2024;14:30147. doi: 10.1038/s41598-024-81734-y

29 Noman SM, Fadel YM, Henedak MT, *et al.* Leveraging survival analysis and machine learning for accurate prediction of breast cancer recurrence and metastasis. *Sci Rep*. 2025;15:3728. doi: 10.1038/s41598-025-87622-3

30 Weiss K, Khoshgoftaar TM, Wang D. A survey of transfer learning. *J Big Data*. 2016;3:9. doi: 10.1186/s40537-016-0043-6

31 Kim HE, Cosa-Linan A, Santhanam N, *et al.* Transfer learning for medical image classification: a literature review. *BMC Med Imaging*. 2022;22:69. doi: 10.1186/s12880-022-00793-7

32 Dhruba SR, Rahman R, Matlock K, *et al.* Application of transfer learning for cancer drug sensitivity prediction. *BMC Bioinformatics*. 2018;19:497. doi: 10.1186/s12859-018-2465-y

33 Wiens J, Guttag J, Horvitz E. A study in transfer learning: leveraging data from multiple hospitals to enhance hospital-specific predictions. *Journal of the American Medical Informatics Association*. 2014;21:699–706. doi: 10.1136/amiajnl-2013-002162

34 Sunilkumar G, Kumaresan P. Deep Learning and Transfer Learning in Cardiology: A Review of Cardiovascular Disease Prediction Models. *IEEE Access*. 2024;12:193365–86. doi: 10.1109/ACCESS.2024.3514093

35 Khan S, Islam N, Jan Z, *et al.* A novel deep learning based framework for the detection and classification of breast cancer using transfer learning. *Pattern Recognition Letters*. 2019;125:1–6. doi: 10.1016/j.patrec.2019.03.022

36 Bellot A, Schaar M van der. Boosting Transfer Learning with Survival Data from Heterogeneous Domains. *Proceedings of the Twenty-Second International Conference on Artificial Intelligence and Statistics*. PMLR 2019:57–65.

37 Menand ES, Jrad N, Marion J-M, *et al.* Predicting clinical outcomes of ovarian cancer patients: deep survival models and transfer learning. 2021.

38 Zhu F, Zhong R, Li F, *et al.* Development and validation of a deep transfer learning-based multivariable survival model to predict overall survival in lung cancer. *Transl Lung Cancer Res*. 2023;12:471–82. doi: 10.21037/tlcr-23-84

39 Kim S, Kim K, Choe J, *et al.* Improved survival analysis by learning shared genomic information from pan-cancer data. *Bioinformatics*. 2020;36:i389–98. doi: 10.1093/bioinformatics/btaa462

40 Zhang Y, Lobo-Mueller EM, Karanicolas P, *et al.* CNN-based survival model for pancreatic ductal adenocarcinoma in medical imaging. *BMC Med Imaging*. 2020;20:11. doi: 10.1186/s12880-020-0418-1

41 Wishart GC, Azzato EM, Greenberg DC, *et al.* PREDICT: a new UK prognostic model that predicts survival following surgery for invasive breast cancer. *Breast Cancer Research*. 2010;12:R1. doi: 10.1186/bcr2464

42 Grootes I, Wishart GC, Pharoah PDP. An updated PREDICT breast cancer prognostic model including the benefits and harms of radiotherapy. *npj Breast Cancer*. 2024;10. doi: 10.1038/s41523-024-00612-y

43 Chen E, Chen C, Chen Y, *et al.* Insights into the performance of PREDICT tool in a large Mainland Chinese breast cancer cohort: a comparative analysis of versions 3.0 and 2.2. *The Oncologist*. 2024;29:e976–83. doi: 10.1093/oncolo/oyae164

44 Hsiao Y-W, Wishart GC, Pharoah PDP, *et al.* Validation of the PREDICT Breast Version 3.0 Prognostic Tool in US Breast Cancer Patients. Published Online First: October 2024. doi: 10.1101/2024.10.29.24316401

45 Basmadjian RB, Xu Y, Quan ML, *et al.* Evaluating PREDICT and developing outcome prediction models in early-onset breast cancer using data from Alberta, Canada. *Breast Cancer Res Treat*. 2025;211:399–408. doi: 10.1007/s10549-025-07654-1

46 Pharoah PDP, Hsiao Y-W, Wishart GC, *et al.* PREDICT breast v4.0: an update to the PREDICT breast prognostic model. *BMC Res Notes*. 2025;18:482. doi: 10.1186/s13104-025-07552-1

47 pengpclab. pengpclab/PREDICTv4.1.1. 2025.

48 Altalhan M, Algarni A, Turki-Hadj Alouane M. Imbalanced Data Problem in Machine Learning: A Review. *IEEE Access*. 2025;13:13686–99. doi: 10.1109/ACCESS.2025.3531662

49 Lunardon N, Menardi G, Torelli N. ROSE: A package for binary imbalanced learning. Published Online First: 2014. doi: 10.32614/rj-2014-008

50 Lunardon N, Menardi G, Torelli N. ROSE: Random Over-Sampling Examples. 2021.

51 Ishwaran H, Kogalur UB. randomForestSRC: Fast Unified Random Forests for Survival, Regression, and Classification (RF-SRC). 2025.

52 Buuren S van, Groothuis-Oudshoorn K, Vink G, *et al.* mice: Multivariate Imputation by Chained Equations. 2024.

53 Chen T, He T, Benesty M, *et al.* xgboost: Extreme Gradient Boosting. 2025.

54 pengpclab/PREDICTv3: This R package provides an updated implementation of the PREDICT Breast cancer prognostication model v3.0. https://github.com/pengpclab/PREDICTv3/tree/main (accessed 8 May 2025)

55 Pilgram L, El Emam K. Transfer Learning and Machine Learning for Breast Cancer Survival Prediction. OSF. 2025. https://doi.org/10.17605/OSF.IO/8N4EP

56 Nelder JA, Mead R. A Simplex Method for Function Minimization. *The Computer Journal*. 1965;7:308–13. doi: 10.1093/comjnl/7.4.308

57 Bolar K. STAT: Interactive Document for Working with Basic Statistical Analysis. 2019.

58 Predict: Breast Cancer. Derivation of the underlying model as implemented in PREDICT v3.0.

59 Goss PE, Ingle JN, Pritchard KI, *et al.* Exemestane versus anastrozole in postmenopausal women with early breast cancer: NCIC CTG MA.27--a randomized controlled phase III trial. *J Clin Oncol*. 2013;31:1398–404. doi: 10.1200/JCO.2012.44.7805

60 Health Canada. Canadian Tobacco Use Monitoring Survey: Smoking in Canada: An Overview. 2003.

61 Breast Cancer HER2 Status | What is HER2 Status? https://www.cancer.org/cancer/types/breast-cancer/understanding-a-breast-cancer-diagnosis/breast-cancer-her2-status.html (accessed 7 May 2025)

62 Chaurasia M, Singh R, Sur S, *et al.* A review of FDA approved drugs and their formulations for the treatment of breast cancer. *Front Pharmacol*. 2023;14:1184472. doi: 10.3389/fphar.2023.1184472

63 Crown J, O'Leary M. The taxanes: an update. *The Lancet*. 2000;355:1176–8. doi: 10.1016/S0140-6736(00)02074-2

64 Piccart MJ, Lohrisch C, Duchateau L, *et al.* Taxanes in the Adjuvant Treatment of Breast Cancer: Why Not Yet? *JNCI Monographs*. 2001;2001:88–95. doi: 10.1093/oxfordjournals.jncimonographs.a003468

65 Maureen E. Trudeau MD. Optimizing Adjuvant Breast Cancer Chemotherapy: Rationale for the MA.21 Study. 2001;15.

66 Burnell M, Levine MN, Chapman J-AW, *et al.* Cyclophosphamide, Epirubicin, and Fluorouracil Versus Dose-Dense Epirubicin and Cyclophosphamide Followed by Paclitaxel Versus Doxorubicin and Cyclophosphamide Followed by Paclitaxel in Node-Positive or High-Risk Node-Negative Breast Cancer. *JCO*. 2010;28:77–82. doi: 10.1200/JCO.2009.22.1077

67 van de Velde CJH, Rea D, Seynaeve C, *et al.* Adjuvant tamoxifen and exemestane in early breast cancer (TEAM): a randomised phase 3 trial. *Lancet*. 2011;377:321–31. doi: 10.1016/S0140-6736(10)62312-4

68 Yan Y. rBayesianOptimization: Bayesian Optimization of Hyperparameters. 2024.

69 Austin PC, Harrell Jr FE, van Klaveren D. Graphical calibration curves and the integrated calibration index (ICI) for survival models. *Statistics in Medicine*. 2020;39:2714–42. doi: 10.1002/sim.8570
