# Supplementary material for: Transfer Learning and Machine Learning for Training Five-Year Survival Prognostic Models in Early Breast Cancer: Development and Validation Study
Source: J Med Internet Res. 2026 Apr 14;28:e88665. doi: 10.2196/88665 (PMC13125978; doi:10.2196/88665)
Supplement: Multimedia Appendix 2 [file jmir_v28i1e88665_app2.pdf]

Consolidated Reporting Guidelines for Prognostic and Diagnostic Machine Learning Models (CREMLS)

From [1]

#	Item	Y	N	NA	Location / Reasoning
**Study Details**					
1.1	*The medical/clinical task of interest*	x			Introduction
1.2	*The research question*	x			Introduction
1.3	*Current medical/clinical practice*	x			Introduction
1.4	*The known predictors and confounders to what is being predicted / diagnosed*	x			Methods – MA.27 Study Cohort,<br>Appendix A: Supplemental Methods – Variable Mapping of MA.27, SEER and TEAM
1.5	*The overall study design*	x			Methods – Data Sources,<br>Methods – Model Training, Testing and Internal Validation Figure 1
1.6	*The medical institutional setting(s)*	x			Methods – Data Sources,<br>Ethics
1.7	*The target patient population*	x			Introduction,<br>Methods – MA.27 Study Cohort,<br>Methods – External Validation
1.8	*The intended use of the ML model*	x			Introduction,<br>Discussion – Implications for Practice
1.9	*Existing model performance benchmarks for this task*	x			Appendix A: Supplemental Background – The Pre-Trained Model PREDICT

					in Breast Cancer Survival Analysis,<br>Results – Performance Across Transfer Learning, De Novo ML, and Ensemble Integration Table 3,<br>Discussion – Summary and Comparison to Literature
1.10	*Ethical and other regulatory approvals obtained*	x			Ethics
**The Data**					
2.1	*Inclusion / exclusion criteria for the patient cohort*	x			Methods – Data Sources,<br>Methods – MA.27 Study Cohort,<br>Methods – External Validation
2.2	*Methods of data collection*	x			Methods – Data Sources
2.3	*Bias introduced due to the method of data collection used*	x			Limitations
2.4	*Data characteristics*	x			Results – Description of MA.27 Study Cohort Table 2,<br>Appendix A: Supplemental Results – Descriptive Characteristics of MA.27
2.5	*Methods of data transformations and preprocessing applied*	x			Methods – Data Management,<br>Appendix A: Supplemental Methods – Outcome Re-Balancing,<br>Appendix A: Supplemental Methods – Missingness Analyses and Model-Based Imputation,

					Appendix A: Supplemental Methods – Variable Mapping of MA.27, SEER and TEAM
2.6	*Known quality issues with the data*	x			Methods – Data Management,<br>Discussion – Relevance of Missing Information,<br>Discussion – Addressing Outcome Imbalance
2.7	*Sample size calculation*			x	We did not conduct formal sample size calculations. Instead, our considerations focused on data sufficiency for training including strategies to address class imbalance (i.e., ROSE and algorithm-level weighting).
2.8	*Data Availability*	x			Data Availability
**Methodology**					
3.1	*Strategies for handling missing data*	x			Methods – Data Management,<br>Appendix A: Supplemental Methods – Missingness Analyses and Model-Based Imputation
3.2	*Strategies for addressing class imbalance*	x			Methods – Data Management,<br>Appendix A: Supplemental Methods – Outcome Re-Balancing
3.3	*Strategies for reducing dimensionality of data*			x	Data was low dimensional with a limited set of clinicopathological predictors.

3.4	*Strategies for handling outliers*	x			Methods – MA.28 Study Cohort
3.5	*Strategies for data augmentation*			x	We applied ROSE as rebalancing strategy to address class imbalance. This is a data augmentation method (oversampling) but not in the sense of creating entirely new synthetic records.
3.6	*Strategies for model pre-training*	x			Methods – Survival Models,<br>Appendix A: Supplemental Methods – Pre-Trained Model and Transfer Learning
3.7	*The rationale for selecting the machine learning algorithm*	x			Introduction,<br>Methods – Survival Models,<br>Appendix A: Supplemental Methods – Machine Learning Survival Models
3.8	*The method of evaluating model performance during training*	x			Methods – Performance Measurement, Decision Curve and Model Explainability,<br>Methods – Model Training, Testing and Internal Validation
3.9	*The method used for hyperparameter tuning*	x			Methods – Model Training, Testing and Internal Validation,<br>Appendix A: Supplemental Methods – Machine Learning Survival Models
3.10	*Model's output adjustments*	x			Methods – Performance Measurement, Decision

					Curve and Model Explainability
**Evaluation**					
4.1	*Performance metrics used to evaluate the model*	x			Methods – Performance Measurement, Decision Curve and Model Explainability
4.2	*The cost or consequence of errors*	x			Methods – Performance Measurement, Decision Curve and Model Explainability
4.3	*The results of internal validation*	x			Results – Performance Across Transfer Learning, De Novo ML, and the Stacked Ensemble Table 3
4.4	*The final model hyperparameters*	x			Appendix A: Supplemental Results – Parameter Variability Across 50 Independent Runs
4.5	*Model evaluation on an external dataset*	x			Results – External Validation on SEER and TEAM,<br>Appendix A: Supplemental Results – External Evaluation Plots
4.6	*Characteristics relevant for detecting data shift and drift*	x			Appendix A: Supplemental Results – Stratified Results from External Validation on SEER,<br>Discussion – Summary and Comparison to Literature,<br>Discussion – Implications for Practice
**Explainability and Transparency**					

5.1	*The most important features and how they relate to the outcome(s)*	x			Results – Model Explainability Figure 4
5.2	*Plausibility of model outputs*	x			Results – Model Explainability Figure 4, Results – Performance Across Transfer Learning, De Novo ML, and the Stacked Ensemble Table 3
5.3	*Interpretation of model's results by an end-user*			x	Model results are expressed in 5-year survival probabilities which are straightforward to interpret for clinicians. We did, however, not explicitly design or test a user interface for clinical use given the methodological scope of this study.

References

[1] W. Klement and K. El Emam, "Consolidated Reporting Guidelines for Prognostic and Diagnostic Machine Learning Modeling Studies: Development and Validation," *J Med Internet Res*, vol. 25, p. e48763, Aug. 2023, doi: 10.2196/48763.
